# A Bayesian Modeling Framework for Health Care Resource Use and Costs in Trial-Based Economic Evaluations

**DOI:** 10.1177/0272989X251376026

**Published:** 2025-10-23

**Authors:** Andrea Gabrio

**Affiliations:** Department of Methodology and Statistics, Faculty of Health Medicine and Life Science, Maastricht University, Maastricht, the Netherlands

**Keywords:** trial-based economic evaluation, cost-effectiveness analysis, health care resource use, Bayesian statistics, missing data

## Abstract

Individual-level data are routinely used in trial-based economic evaluations to assess the effectiveness and costs of a given intervention. While effectiveness measures are often expressed via utility scores derived from health-related quality-of-life instruments (e.g., EQ-5D questionnaires), information on different types of health care resource use (HRU) measures (e.g., number and types of services) are collected to compute the costs. Partially complete HRU data, particularly for self-reported questionnaires, are handled via ad hoc methods that rely on some assumptions (fill in a zero) that are typically hard to justify. Although methods have been proposed to account for the uncertainty surrounding missing data, particularly in the form of multiple imputation or Bayesian methods, these have mostly been implemented at the level of costs at different times or over the entire study period, while little attention has been given to how missing values at the level of HRUs should be addressed and their implications on the final analysis. We present a general Bayesian framework for the analysis of partially observed HRUs in trial-based economic evaluations, which can accommodate the typical complexities of the data (e.g., excess zeros, skewness, missingness) and quantify the impact of missingness uncertainty on the results. We show the benefits of our approach with a motivating example and compare the results to those from more standard analyses fitted at the level of cost variables after adopting some ad hoc imputation. This article highlights the importance of adopting a comprehensive modeling approach to handle partially observed HRU data in economic evaluations and the strategic advantages of building these models within a Bayesian framework.

**Highlights:**

- Missing health care service data in trial-based economic evaluations are often removed or imputed using quite restrictive assumptions (e.g., no use of service).
- We propose a flexible Bayesian approach to account for missing health care service uncertainty and compare the results with models fitted at more aggregated levels (e.g., total costs) using a real case study.
- Our results show that, depending on the (assumed) missingness assumptions and the level of data aggregation at which analyses are performed, results may be considerably changed.
- When feasible, analyses should be conducted at the most disaggregated level to ensure that all available information collected in the trial is used in the analysis without relying on (often) restrictive ad hoc imputation approaches.

Economic evaluations represent an increasingly important component in the process of health technology assessment (HTA) of new health care interventions in many countries.^1,2^ In the context of trial-based economic evaluations, HTA institutions recommend cost-utility analysis as the “reference case” approach, using health-related quality-of-life (HRQol) utility scores as the health outcome to ensure direct comparability of interventions across disease areas. Alongside utilities, costs are computed by applying the appropriate unit prices, retrieved from the literature or national sources, to the amount of health care resource use (HRU) consumed by the patients over a certain period of time (e.g., number and type of visits over the past 3 mo). Self-reported questionnaires, such as the Client Service Receipt Inventory^3^ and iMedical Cost Questionnaire,^4^ are routinely used to collect HRU data but often suffer from low completion rates.

Previous reviews have advocated for the importance of dealing with missing outcome data in trial-based economic evaluations^5,6^ and have highlighted an overall improvement over time in current practice toward the adoption of approaches that formally take into account the uncertainty around missing values, such as multiple imputation.^7^ A more recent review provided an updated analysis of the missingness approaches used in economic evaluations and revealed a somewhat unclear picture about the consistency of the methods used to address missingness at different levels of aggregation.^8^ More specifically, the authors highlighted a clear difference between the typical methods used to handle missing values at the level of cost/utility data, where multiple imputation is often used, compared with the level of questionnaire items, where some form of single imputation approach is used. While this may not pose a problem for EQ-5D questionnaires, often characterized by unit nonresponse (all answers skipped), the same cannot be said for HRU questionnaires, which are typically lengthy and subject to item nonresponse (some answers skipped). As a result, when confronted with partially missing HRU responses, analysts may decide to “fill in the gaps” based on some specific assumptions such as no use of service (i.e., imputed as 0). Although this can be justified in some cases based on external information, when this is lacking, it is essential that the uncertainty around missing HRU data is properly quantified and its impact on the analysis results reflected.

Building on the existent literature, we propose a novel Bayesian framework for trial-based economic evaluations that allows a flexible model specification and the handling of missing data at the HRU level. The choice of a Bayesian approach has practical advantages compared with a standard frequentist framework,^9,10^ including 1) the use of a modular structure to increase model complexity in a relatively easy way,^11^ 2) natural interpretation of cost-effectiveness results in probabilistic terms,^12^ and 3) direct implementation of probabilistic sensitivity analysis, consisting in the quantification of the impact of parameters’ uncertainty on the conclusions.^13^ We show the benefits of using our framework on a real case study, with a focus on appropriately modeling partially observed HRU values and its implications in terms of inferences and, crucially, cost-effectiveness results.

The article is structured as follows: the second section presents the case study and describes the data. The third section defines the general framework of the statistical models used to analyze costs/utilities and HRU values. The fourth section compares the results of models fitted at different levels of aggregation and shows how different assumptions about partially missing cases may affect the model estimates. The fifth section performs the economic evaluation, summarizes the inferences for each model, and compares the cost-effectiveness results. The sixth section discusses the proposed framework and suggests some improvements for future work. Finally, the Appendix includes additional material related to model assessment and results, while the software code is provided in the online supplementary material.

## Case Study: The PBS Trial

The Positive Behaviour Support (PBS)^14^ trial was a multicenter, randomized controlled trial involving community intellectual disability services and service users with mild to severe intellectual disabilities and challenging behaviors. Positive behavior support is a multicomponent intervention that is designed to foster prosocial actions and enhance the person’s quality of life and their integration within the local community. Participants (*N = 244*) were enrolled and randomly allocated to staff teams trained to deliver PBS in addition to treatment as usual (reference intervention, $n_{2}=108$ ) or to staff teams trained to deliver treatment as usual alone (comparator, $n_{1}=136$ ). Measures for quality of life in the form of EQ-5D-3L questionnaires^15^ and HRU information based on Client Service Receipt Inventory (CSRI) questionnaires^3^ were collected for each individual ( i=1,…,n ) at baseline ( j=0 ) and at 6 and 12 ( j=1,2 ) mo of follow-up.

Individual-level utility scores at each time $u_{\mathrm{ij}}$ were computed based on the patients’ answers to the EQ-5D questionnaires and national value sets,^16^ while HRU data were collected through CSRI questionnaires and covered a wide range of health care services. For the purpose of this study, we consider individual-level HRUs collected at each time ${\mathrm{HRU}}_{\mathrm{ij}}^{k}$ on k=1,…,K=9 services: number of visits with a psychiatric doctor (PSYDR), psychologist (PSYCH), physiotherapist (PHYSI), dentist (DENT), social worker (SOCWORK), community worker (COMWORK), general practitioner (GP), nurse (NURSE), and private therapist (THERAP). CSRI data were characterized both by unit and item nonresponse, meaning that individuals may either provide full or only partial information in terms of the services used at each time. A statistical summary of the missingness rates of the utility and HRU variables by time point in the PBS trial is shown in Table 1.

**Table 1:** Number (Proportion) of Missing Cases for the Utilities and Different Health Care Resource Use Services (Self-Reported Questionnaires), Presented by Time Point of the Trial^a^

Outcome	Baseline (*j* = 0)	6 mo ( j=1 )	12 mo ( j=2 )	ic (Outcome)
Utilities ( u )	14 (5.7%)	23 (9.4%)	16 (6.6%)	40 (16.4%)
PSYDR ( $\mathrm{hr}u^{1}$ )	0	18 (7.4%)	12 (4.9%)	22 (9%)
PSYCH ( $\mathrm{hr}u^{2}$ )	2 (1.4%)	12 (4.9%)	9 (3.7%)	14 (5.7%)
PHYSI ( $\mathrm{hr}u^{3}$ )	1 (0.4%)	13 (5.3%)	10 (4.1%)	16 (6.6%)
DENT ( $\mathrm{hr}u^{4}$ )	1 (0.4%)	14 (5.7%)	12 (4.9%)	19 (7.8%)
SOCWORK ( $\mathrm{hr}u^{5}$ )	1 (0.4%)	14 (5.7%)	11 (4.5%)	18 (7.4%)
COMWORK ( $\mathrm{hr}u^{6}$ )	0	13 (5.3%)	11 (4.5%)	15 (6.1%)
GP ( $\mathrm{hr}u^{7}$ )	3 (2.1%)	15 (6.2%)	14 (5.7%)	22 (9%)
NURSE ( $\mathrm{hr}u^{8}$ ) 2	(1.4%)	17 (7%)	13 (5.3%)	24 (9.8%)
THERAP ( $\mathrm{hr}u^{9}$ )	1 (0.4%)	15 (6.1%)	12 (4.9%)	19 (7.8%)
ic (time)	10 (4.2%)	22 (9%)	17 (7%)	59 (24.2%)

a In the table, the number (%) of individuals with partially observed data at each time across outcomes (bottom row), for each outcome across times (rightmost column), and across both times and outcomes (bottom right cell) is also reported. At each time *j*, only unit nonresponse characterizes EQ-5D questionnaires (and so utilities), while both unit and item nonresponse affects Client Service Receipt Inventory questionnaires (and so health care resource uses).

Overall, the proportions of missing values are moderate and never exceed 10% at any time point. However, when aggregating all variables, missingness becomes more substantial, with a proportion of incomplete cases of ≈24% (bottom right cell in Table 1).

In addition to missingness, a typical feature of HRU data is the amount of “extreme” values that remain constant over time (e.g., zero values), typically denoted as “structural values.”^17^ Table 2 reports the number (proportions) of structural zeros observed for each type of HRU service in the PBS study.

**Table 2:** Number (Proportion) of Structural Zeros Observed for Each Type of HRU Service in the PBS Study

Outcome	Structural Zeros
PSYDR ( $\mathrm{hr}u^{1}$ )	37 (15%)
PSYCH ( $\mathrm{hr}u^{2}$ )	159 (65%)
PHYSI ( $\mathrm{hr}u^{3}$ )	189 (77%)
DENT ( $\mathrm{hr}u^{4}$ )	35 (14%)
SOCWORK ( $\mathrm{hr}u^{5}$ )	47 (19%)
COMWORK ( $\mathrm{hr}u^{6}$ )	196 (80%)
GP ( $\mathrm{hr}u^{7}$ )	1 (0.4%)
NURSE ( $\mathrm{hr}u^{8}$ )	53 (22%)
THERAP ( $\mathrm{hr}u^{9}$ )	85 (35%)

HRU, health care resource use; PBS, Positive Behaviour Support.

The proportions of structural zeros in most HRUs range from 15%to80% , with the only exception being the number of GP visits ( 0.4% ). The presence of these zero values induces a high degree of skewness in each of these variables’ empirical distributions, which needs to be appropriately addressed in the analysis stage.

## Modeling Framework

We first present our framework building upon previously introduced approaches. We start by considering models fitted at the level of total costs and quality-adjusted life-years (QALYs)^18−21^ or costs and utilities at different times^22,23^ and then extend these to account for partially observed HRU data. The framework allows for typical features of cost-effectiveness data (e.g., skewness, correlation, structural values) while also handling missing HRUs without relying on ad hoc assumptions (e.g., assumed zeros). Although different study designs in terms of data collection could be considered (e.g., multiple times for the effectiveness but only 1 for costs), we argue that the PBS data reflect the typical design used in many trial-based economic evaluations.^24^ Thus, we illustrate the framework using the trial design (see the second section) as reference, where disaggregated outcomes are collected at baseline and equally spaced follow-up times. However, we note that the framework can also be modified to handle different types of data structures, including different collection times between outcomes and/or a cross-sectional design for some variables.

Assume that some patient-level HRU and HRQol data from a trial are collected via self-reported questionnaires (e.g., CSRI and EQ-5D) at equally spaced times j=1,…,J on i=1,…,n individuals, who are randomly allocated to either a control ( t=0 ) or intervention ( t=1 ) group with sample sizes $n_{1}$ and $n_{2}$ , respectively. For each individual and time point, questionnaire answers on multiple health care services and HRQol domains are collected. Let ${\mathrm{HRU}}_{\mathrm{ij}}^{k}$ denote the health resource use information collected on service k (for k=1,…K ) for the i -th individual and j -th time point in the trial, and let ${\mathrm{HrQol}}_{\mathrm{ij}}^{l}$ denote the corresponding HRQol information collected on domain l (for l=1,…,L ). HRU and HRQol data are then, respectively, combined with national unit prices for each type of service $p_{k}$ and value sets to derive service-specific costs $c_{\mathrm{ij}}^{k}$ and utility scores $u_{\mathrm{ij}}$ for each individual at each time in the study, with the total individual costs obtained as the sum of all service-specific costs at each time, that is, $c_{\mathrm{ij}}=\sum_{k=1}^{K}c_{\mathrm{ij}}^{k}$ . Next, total costs ( $tc_{i}$ ) and QALYs ( $e_{i}$ ) for each individual over the trial period are computed as

(1) $$\begin{array}{ccc} tc_{i}=\sum_{j=1}^{J}c_{\mathrm{ij}} & \mathrm{and} & e_{i}=\sum_{j=1}^{J}\left(u_{\mathrm{ij}}+u_{\mathrm{ij}-1}\right)\frac{\delta_{j}}{2}, \end{array}$$

where $\delta_{j}=\frac{{\mathrm{Time}}_{j}-{\mathrm{Time}}_{j-1}}{\mathrm{Unit}\;\mathrm{of}\;\mathrm{time}}$ is the proportion of the time unit (typically 1 y) that is covered between time j−1 and j in the trial.

The framework allows the modeling of different types of variables: HRU and HRQoL, costs and utilities at each time, or total costs and QALYs. In principle, it is arguably easier to specify a model for aggregated (e.g., total costs) compared with disaggregated variables (e.g., costs or HRU), since it allows for performing the analysis based on a fewer number of variables and a cross-sectional setting. However, in reality, the occurrence of missing values at the most disaggregated level (i.e., HRU and HRQoL) is unavoidable, with proportions of unobserved values that are usually substantial.^5,6^ In the presence of partially missing questionnaire answers, modeling at aggregated levels (e.g., costs or total costs) does not allow direct incorporation of the evidence from the partially observed items, which need to be discarded unless their values are imputed prior to the analysis. While item nonresponse is often not a problem for HRQoL,^25^ it severely affects HRU data, with many individuals providing information about the usage of only some of the services.^26^ To our knowledge, guidelines on the handling of partially missing HRU data are not available, with analysts often not reporting detailed information about their proportions of missing values.^8^ When information on HRUs is lacking, it is plausible to assume that some deterministic imputation of item nonresponses is used to improve data completeness. A typical example occurs when individuals with a missing value on a specific service are assumed to not have used it, thus suggesting the replacement of the unobserved value with a zero. However, such assumption can never be verified based on the observed data and, when repeatedly applied for different services, times, and individuals, may have a substantial effect on the analysis results.

We separately present our approach by level of data aggregation: 1) total costs and QALYs $\left(e_{i},tc_{i}\right)$ , 2) costs and utilities computed at each time $\left(u_{\mathrm{ij}},c_{\mathrm{ij}}\right)$ , and 3) HRUs and utilities computed at each time $\left(u_{\mathrm{ij}},{\mathrm{HRU}}_{\mathrm{ij}}^{k}\right)$ . For each level of aggregation, we show how the model can be specified and compared with simpler approaches to handle partially missing cases prior to model fitting. For illustrative purposes, and given that item nonresponse is unlikely to affect HRQoL questionnaires, in the reminder we will assume that item nonresponses affect only HRU data.

### Modeling of Total Costs and QALYs

**Figure 1:**
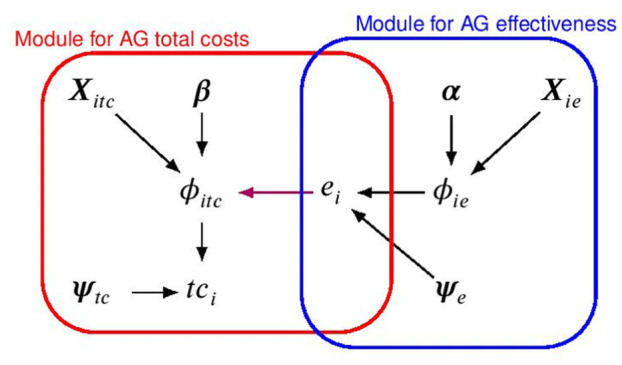
Joint distribution, expressed in terms of a marginal distribution for the effectiveness and a conditional distribution for the total costs, respectively, indicated with a blue and red line. The parameters indexing the corresponding distributions or “modules” are indicated with different Greek letters, while the black and magenta arrows show the dependence relationships between the parameters within and between the 2 models, respectively. Note that i denotes the individual index, while the treatment index t is omitted to ease notation.

At the most aggregated level, data are summarized into effectiveness and total costs ( $e_{i},tc_{i}$ ), which are computed over the study period using equation 1. Most of the methodological literature has focused on the modeling of these variables with an emphasis on the need to specify flexible models to handle the typical complexities of the data. These include correlation between the outcomes, skewness, and presence of structural values.^20,21,27–30^ For instance, dependence between outcomes can be captured by specification of the joint distribution $p\left(e_{i},tc_{i}\right)$ as

(2) $$p\left(e_{i},tc_{i}\right)=p\left(e_{i}\right)p\left(tc_{i}|e_{i}\right)=p\left(tc_{i}\right)p\left(e_{i}|tc_{i}\right),$$

where, for example, $p\left(e_{i}\right)$ is the marginal distribution of the effectiveness and $p\left(tc_{i}|e_{i}\right)$ is the conditional distribution of the total costs given the effectiveness. Note that, while it is possible to use interchangeably either factorization in equation 2, without loss of generality, we describe our analysis in the following through a marginal distribution for the effectiveness and a conditional distribution for the costs. Choice of the distribution for $e_{i}$ and $tc_{i}$ can be guided by the specific characteristics of the data, for example, gamma or lognormal to handle skewness, with each distribution being indexed by a set of location $\left(\phi_{\mathrm{ie}},\phi_{\mathrm{itc}}\right)$ and nuisance $\left(\psi_{e},\psi_{\mathrm{tc}}\right)$ parameters. A generalized linear structure based on some link functions g(·) , such as log or logit, is often used to incorporate outcome-specific covariates at the location level:

(3) $$\begin{array}{ccc} g_{e}\left(\phi_{\mathrm{ie}}\right)=\alpha_{0}+\alpha_{1}X_{\mathrm{ie}} & \mathrm{and} & g_{\mathrm{tc}}\left(\phi_{\mathrm{itc}}\right)=\beta_{0}+\beta_{1}X_{\mathrm{itc}}, \end{array}$$

where the sets of regression parameters α and β include the intercept and covariate-specific coefficients ( $X_{\mathrm{ie}},X_{\mathrm{itc}}$ ) for each model. Posterior estimates of interest, that is, the mean incremental QALYs ( $\Delta_{e}=\mu_{e0}-\mu_{e1}$ ) and total costs ( $\Delta_{\mathrm{tc}}=\mu_{\mathrm{tc}0}-\mu_{\mathrm{tc}1}$ ) between treatment groups, can be derived in terms of linear combinations of the regression parameters or through simulation approaches such as Monte Carlo methods.^9,10^ Figure 1 shows a graphical representation of the modeling framework for the aggregated variables, where the effectiveness and total cost distributions are represented in terms of combined “modules”—the blue and the red boxes—in which the random quantities are linked through logical relationships.

An appealing feature of this strategy is the need to specify only 2 distributions. However, when HRU nonresponse occurs, analysts need to handle the partially observed disaggregated data prior to fitting the model. As a simple solution, they could either discard all cases with partially observed HRUs or impute as zero all HRU nonresponses ( ${\bar{\mathrm{HRU}}}_{\mathrm{ij}}^{k}=0$ ) and use these to generate costs at each time $c_{\mathrm{ij}}$ . In the second case, analysts must also decide how to handle missing cases at the cost level by either discarding all cases with partially observed costs at any time or impute as zero all partially observed costs at each time ( ${\bar{c}}_{\mathrm{ij}}=0$ ) and use these to generate total costs $tc_{i}$ .

### Modeling of Costs and Utilities at Each Time

**Figure 2:**
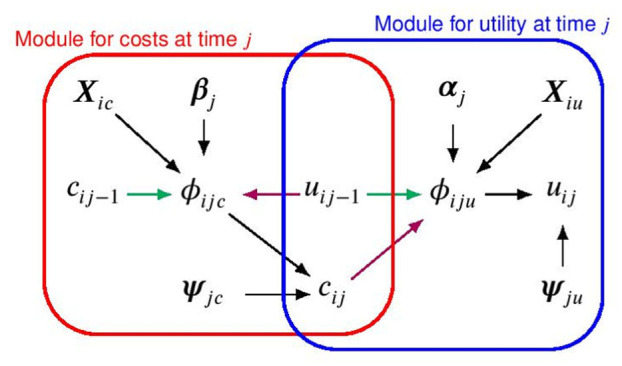
Joint distribution, expressed in terms of a conditional distribution for utilities and costs at time j given their values at time j−1 , respectively, indicated with a blue and red boxes. The parameters indexing the corresponding distributions or “modules” are indicated with different Greek letters, while the black, magenta, and green arrows show the dependence relationships between the parameters within and between the models and between times, respectively. Note that i and j denote the individual and time index, while the treatment index t is omitted to ease notation.

At an intermediate aggregation level, the data consist of 2 longitudinal variables, namely, the utilities and costs computed at each time ( $u_{\mathrm{ij}},c_{\mathrm{ij}}$ ). In recent years, attention has been given to the specification of longitudinal models that can deal with missing outcomes at different times while also addressing the complexities of the data.^22,23^ For example, the joint outcome distribution $p\left(u_{\mathrm{ij}},c_{\mathrm{ij}}\right)$ at time j>0 can be specified as

(4) $$p\left(u_{\mathrm{ij}},c_{\mathrm{ij}}\right)=p\left(u_{\mathrm{ij}}|u_{i,j-1},c_{\mathrm{ij}}\right)p\left(c_{\mathrm{ij}}|u_{\mathrm{ij}-1},c_{i,j-1}\right),$$

where $p\left(u_{\mathrm{ij}}|u_{i,j-1},c_{\mathrm{ij}}\right)$ is the conditional distribution of the utilities at j>0 given utilities at time j−1 and costs at j , while $p\left(c_{\mathrm{ij}}|u_{\mathrm{ij}-1},c_{\mathrm{ij}-1}\right)$ is the conditional distribution of the costs at j>0 given utilities and costs at j−1 . Similarly to the modeling of aggregated variables, distributions for $u_{\mathrm{ij}}$ and $c_{\mathrm{ij}}$ are indexed by a set of time-specific location $\left(\phi_{\mathrm{iju}},\phi_{\mathrm{ijc}}\right)$ and nuisance $\left(\psi_{\mathrm{ju}},\psi_{\mathrm{jc}}\right)$ parameters and should be chosen according to the specific features of the data. When a generalized linear structure based on some link functions $g_{u}\left(\cdot \right)$ and $g_{c}\left(\cdot \right)$ is used to incorporate outcome-specific covariates at the location level, the conditional mean at time j for the utilities and costs can be expressed as

(5) $$\begin{array}{ccc} g_{u}\left(\phi_{\mathrm{iju}}\right)=\alpha_{j0}+\alpha_{j1}X_{\mathrm{iu}} & \mathrm{and} & g_{c}\left(\phi_{\mathrm{ijc}}\right)=\beta_{j0}+\beta_{j1}X_{\mathrm{ic}}, \end{array}$$

where the sets of regression parameters α and β include the time-specific intercept and covariate-related coefficients for each model. Once estimates for the marginal mean utility and cost for each treatment group and time ( $\mu_{\mathrm{ju}},\mu_{\mathrm{jc}}$ ) are retrieved from the model, posterior estimates at the levels of QALYs and total costs ( $\Delta_{e},\Delta_{\mathrm{tc}}$ ) can be derived based on $\mu_{\mathrm{ju}}$ and $\mu_{\mathrm{jc}}$ and equation 1. Figure 2 shows a graphical representation of the modeling framework for the utility and cost distributions at time j in terms of combined modules denoted by the blue and red boxes, respectively.

Although this strategy can be more challenging to implement than that in the “Modelling of Total Costs and QALYs” section, it provides a more efficient way to handle partially observed cases. Indeed, by modeling longitudinal variables, missingness uncertainty at any time can be directly quantified through a probabilistic approach without relying on ad hoc imputation methods. However, if participants provide partial information at the level of the questionnaires (i.e., item nonresponse), analysts are still required to handle partially observed responses prior to model fitting. As a simple solution, they could either discard all cases with partially observed HRUs or impute as zero all partially observed HRUs at each time ( ${\bar{\mathrm{HRU}}}_{\mathrm{ij}}^{k}=0$ ) and use these to generate costs at each time $c_{\mathrm{ij}}$ .

### Modeling of HRU Services and Utilities at Each Time

At the most disaggregated level, the data consist in the series of longitudinal variables ${\mathrm{hrqol}}_{\mathrm{ij}}^{l},{\mathrm{hru}}_{\mathrm{ij}}^{k}$ , corresponding to the HRU and HRQoL individual responses at each time provided to the k -th and l -th item of the questionnaires, for k=1,…,K and l=1,…,L . Given that HRQoL questionnaires are mostly affected by unit nonresponse, no substantial gain can be obtained by focusing the modeling at the level of the items compared with the utilities,^31^ whereas HRU questionnaires are often characterized by item nonresponse. As a result, we can specify the model at the level of $u_{\mathrm{ij}}$ and ${\mathrm{hru}}_{\mathrm{ij}}^{k}$ to make full use of the available information in the study. For example, the joint outcome distribution $p\left(u_{\mathrm{ij}},{\mathrm{hru}}_{\mathrm{ij}}^{k}\right)$ at time j>0 can be specified as

(6) $$p\left(u_{\mathrm{ij}},{\mathrm{hru}}_{\mathrm{ij}}^{k}\right)=p\left(u_{\mathrm{ij}}|u_{i,j-1},{\mathrm{hru}}_{\mathrm{ij}}^{k}\right)p\left({\mathrm{hru}}_{\mathrm{ij}}^{k}|u_{\mathrm{ij}-1},{\mathrm{hru}}_{i,j-1}^{k}\right),$$

where $p({\mathrm{hru}}_{\mathrm{ij}}^{k}|u_{\mathrm{ij}-1},{\mathrm{hru}}_{\mathrm{ij}-1}^{k})$ is the conditional distribution of the k -th service at j>0 given utilities and the past HRU value at j−1 . Similarly to the modeling of $c_{\mathrm{ij}}$ , the distributions of ${\mathrm{hru}}_{\mathrm{ij}}^{k}$ are indexed by the set of time and service-specific location $\left(\phi_{\mathrm{ijhru}}^{k}\right)$ and nuisance $\left(\psi_{\mathrm{jhru}}^{k}\right)$ parameters and should be chosen to handle the specific features of the data. While the generalized linear model structure for longitudinal outcomes in the “Modeling of Costs and Utilities at Each Time” section can also be applied to HRU data, model parameters should be given appropriate interpretations. For example, some link function $g_{\mathrm{hru}}\left(\cdot \right)$ can be used to incorporate HRU-specific covariates ( $X_{\mathrm{ihru}}$ ) at the level of the conditional usage rate for service k at time j :

(7) $$g_{\mathrm{hru}}\left(\phi_{\mathrm{ijhru}}^{k}\right)=\beta_{j0}^{k}+\beta_{j1}^{k}X_{\mathrm{ihru}},$$

where the sets of regression parameters β include the time- and service-specific intercept and covariate-related coefficients for the model. An additional complication is that service-specific HRUs are often characterized by large proportions of zero values. To overcome this problem, the use of 2-part regressions or Hurdle models has been suggested in the literature, although it has mostly been applied to cost variables.^20^ These consist in mixture models defined by 2 components: the first is a mass distribution at the spike, while the second is a parametric model applied to the natural range of the relevant variable. Typically, a logistic regression is used to estimate the probability of incurring a “structural” value (e.g., 0 ); this is then used to weight the mean of the “nonstructural” values estimated in the second component.

To specify a Hurdle model for HRUs, we first define an individual indicator variable $d_{\mathrm{ihru}}^{k}$ taking value 1 if the i− th individual is associated with a zero value on the k -th service at all times ( ${\bar{hru}}_{i\forall j}^{k}=0$ ) and 0 otherwise ( ${\mathrm{hru}}_{i\forall j}^{k}>0$ ). This is then modeled as

(8) $$\begin{array}{c} d_{\mathrm{ihru}}^{k}:=I\left({\bar{\mathrm{hru}}}_{i\forall j}=0\right)\sim \mathrm{Bernoulli}\left(\eta_{i}^{k}\right)\\ \mathrm{logit}\left(\eta_{i}^{k}\right)=\gamma_{0}^{k}+\gamma_{1}^{k}X_{i0}, \end{array}$$

where $\eta_{i}^{k}$ is the conditional probability of a zero structural value in the k -th service, which is estimated on the logit scale as a function of a set of regression parameters γ and covariates $X_{i0}$ . Estimates for the marginal probability of a structural zero $\pi^{k}$ can be obtained either by linear combination of the regression parameters or by simulation based on equation 8. Estimates for the marginal usage rate of nonzero HRU values $\mu_{j>0}^{k}$ can be obtained by fitting equation 7 to only nonzero HRUs. Next, we compute the overall population average HRU usage rate for each service and time $\mu_{\mathrm{jhru}}^{k}$ as the linear combination

$$\mu_{\mathrm{jhru}}^{k}=\left(1-\pi^{k}\right)\mu_{j>0}^{k}.$$

**Figure 3:**
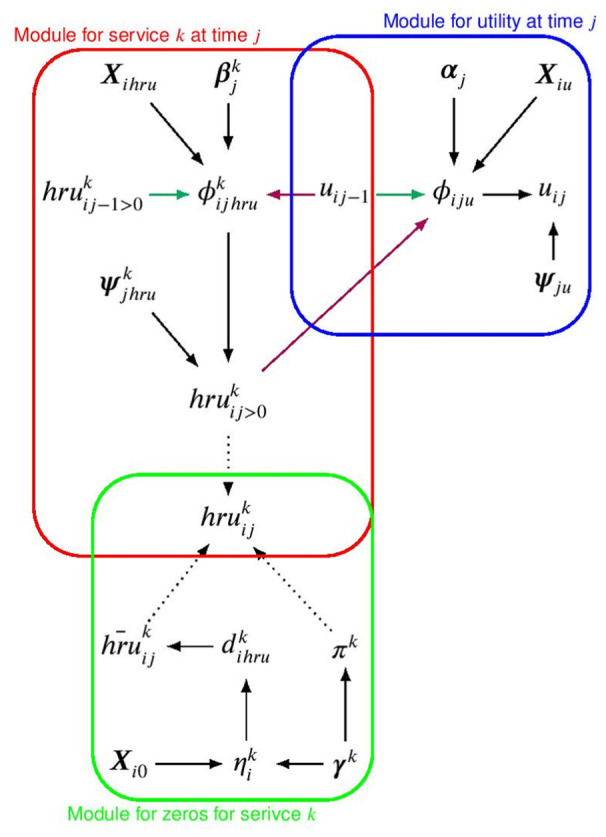
Joint distribution, expressed in terms of conditional distributions for utilities, nonzero and structural zero HRUs at time j given outcomes at time j−1 , respectively, indicated with a blue, red, and green boxes. The parameters indexing the corresponding distributions or “modules” are indicated with different Greek letters, while black, magenta, and green solid arrows show the dependence relationships between the parameters within and between the models and times, respectively. The dashed arrows denote deterministic relationships. Note that i and j denote the individual and time index, while the treatment index t is omitted to ease notation.

These quantities can then be combined with unit prices $p^{k}$ associated with each service k to generate corresponding estimates of marginal mean costs $\mu_{\mathrm{jc}}^{k}$ and summed across all services and times to obtain estimates of the marginal mean total costs $\mu_{\mathrm{tc}}$ . Figure 3 shows a graphical representation of the modeling framework for the utility, non-zero and zero cost distributions at time *j* in terms of modules denoted by the blue, red and green boxes, respectively.

The proposed strategy is more challenging to implement compared with those given in the “Modeling of Total Costs and QALYs” and “Modeling of Costs and Utilities at Each Time” sections due to the presence of multiple HRU variables, each consisting of a 2-part mixture for the nonzero and structural zero component. However, defining the model at the most disaggregated level provides the most efficient way to handle partially observed HRUs. We note that the dependence structure in equation 4 and equation 6 is simply one possible choice, which was selected based on the comparison of the model fit to the PBS data and with respect to alternative specifications. In general, we recommend that analysts explore different specifications and select the one that provides the best compromise between flexible dependence structures between the modeled variables and a feasible implementation to the available data.

## Results

All models were fitted using JAGS,^32^ a software specifically designed for the analysis of Bayesian models using Markov chain Monte Carlo (MCMC) simulation,^33^ which can be interfaced with R through the package R2jags.^34^ Samples from the posterior distribution of the parameters of interest generated by JAGS and saved to the R workspace were then used to produce summary statistics and plots. We ran 2 chains with 20,000 iterations per chain, using a burn in of 10,000 , for a total sample of 20,000 iterations for posterior inference. For each unknown quantity in the model, we assessed convergence and autocorrelation of the MCMC simulations using diagnostic measures such as the potential scale reduction factor and the effective sample size.^35^ For each modeling approach, the fit of different distributions for the HRU/cost variables was compared using standard measures such as the widely applicable information criterion, or WAIC,^36^ and posterior predictive checks. Based on these comparisons, normal distributions for QALYs and utility variables, gamma distributions (compared with normal and lognormal distributions) for all cost variables, and normal distributions (compared with Poisson and negative binomial) for all HRU variables, using a hurdle approach to handle structural zeros was selected as the best-fitting distributions. Alternative prior distributions were considered to check that any unintended information was not incorporated into the models through the priors.

We also compared the results from our analyses to those of a traditional modeling approach based on a frequentist statistical framework. Although it is difficult to identify a standard approach for handling missing values and modeling health economic data in routine analyses, we used information collected from previous reviews^5,6,8^ to identify the methods used in this analysis. A detailed description of the methods used under the traditional modeling approach is provided in Appendix A alongside a summary of the statistical and health economic results derived from it. In Appendix B, we explain in detail how the hurdle model was implemented in JAGS, while in the online supplementary material, we provide the full JAGS code for the models.

### Model Estimates

Table 3 compares the posterior results of models fitted to the PBS data at the level of S1) total costs and QALYs, S2) costs and utilities at each time, and S3) HRU categories and utilities at each time. Within the first 2 strategies, alternative approaches to handle partially observed data are implemented. For strategy 1, missing cases are either included in the analysis (ALL), imputed as zero only for HRUs (IMP-H), or imputed as zero for both HRUs and costs (IMP-HC). For strategy 2, missing cases are either included in the analysis (ALL) or imputed as zero only for HRUs (IMP-H). Finally, for strategy 3, all data with no ad hoc imputation are used (ALL). Table 4 shows the posterior estimates and 95% credible intervals of the marginal mean total costs and QALYs by treatment group ( $\mu_{\mathrm{tc}},\mu_{e}$ ) obtained from the different modeling strategies. Estimates (and credible intervals) for mean costs, utilities, and HRU rates, which can be obtained only from models fitted under strategy 2 and/or 3, are reported in Appendix A.

**Table 3:** Posterior Means and 95% Credible Intervals of $\mu_{\mathrm{tc}}$ and $\mu_{e}$ in the Control ( t=1 ) and Intervention ( t=2 ) Groups Obtained under Alternative Missingness Approaches When Fitting the Model: at the Level of Total Costs and QALYs (Strategy 1), at the Level of Costs and Utilities at Each Time (Strategy 2), and at the Level of HRU and Utilities at Each Time (Strategy 3)

Approach	$\mu_{\mathrm{tc}\left(t=1\right)}$		$\mu_{\mathrm{tc}\left(t=2\right)}$		$\mu_{e\left(t=1\right)}$		$\mu_{e\left(t=2\right)}$	
	Mean	95% CI	Mean	95% CI	Mean	95% CI	Mean	95% CI
Strategy 1: Total cost and QALY								
ALL	2,543	(2,157; 2,938)	2,754	(2,249; 3,310)	0.487	(0.449; 0.575)	0.609	(0.569; 0.649)
IMP-H	2,888	(2,411; 3,393)	2,379	(1,939; 2,872)	0.488	(0.45; 0.526)	0.61	(0.571; 0.651)
IMP-HC	2,395	(1,897; 2,899)	2,237	(1,175; 2,749)	0.486	(0.449; 0.523)	0.61	(0.572; 0.649)
Strategy 2: Cost and utility at each time								
ALL	2,607	(2,253; 2,971)	2,701	(2,278; 3,145)	0.494	(0.463; 0.527)	0.6	(0.566; 0.635)
IMP-H	2,453	(2,087; 2,843)	2,273	(1,874; 2,664)	0.494	(0.462; 0.526)	0.6	(0.565; 0.633)
Strategy 3: HRU category and utility at each time								
ALL	2687	(2,173; 3,194)	2,587	(1,995; 3,206)	0.513	(0.475; 0.55)	0.599	(0.565; 0.634)

HRU, health care resource use; QALY, quality-adjusted life-year.

In general, estimates of mean QALYs in both treatment groups are always very similar. This is expected in that, within each strategy, zero imputation was considered only for HRU/cost variables, thus not affecting utilities and/or QALYs. In addition, since only unit nonresponse affects HRQoL data, no substantial information gain is obtained by modeling utilities compared with QALYs, with average estimates remaining almost unchanged across all strategies.

**Figure 4:**
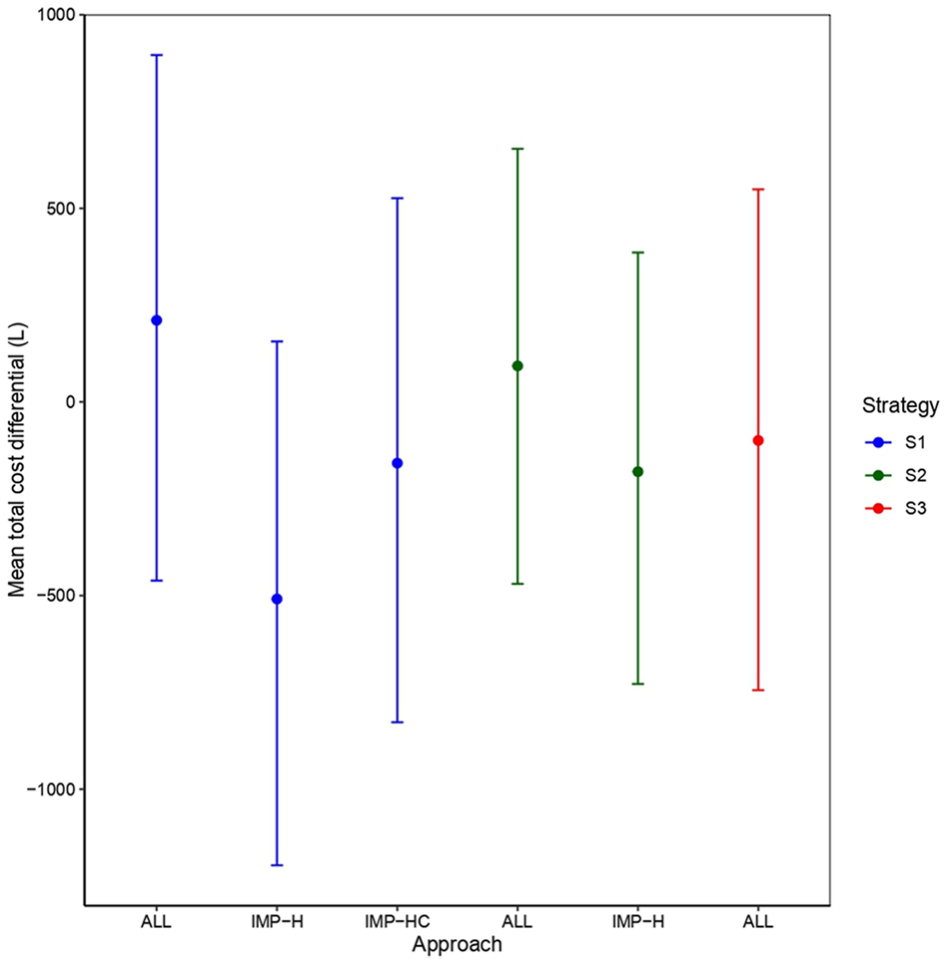
Estimated mean total cost difference between treatment groups in the Positive Behaviour Support study based on different modeling strategies (S1, S2, S3) and alternative approaches to handling missing health care resource use and/or cost data.

A comparison of the estimates obtained under strategy 1 suggests that an analysis fitted to all cases without zero imputation (ALL) is associated with mean total cost estimates for the intervention that are higher compared with those of the control group ( ≈211 £), whereas after zero imputing the data (IMP-H or IMP-HC), the situation is reversed. A similar trend is also observed when comparing the estimates obtained under strategy 2, with the intervention being on average more expensive than the control under ALL ( ≈94 £) but less expensive under IMP-H ( ≈−180 £). Finally, mean estimates obtained under ALL from strategy 3 suggest that the intervention is cheaper than the control ( ≈−100 £) with comparable credible interval widths to those from strategy 2. Figure 4 shows the mean differences in total costs between treatments derived from each model, distinguished according to the type of strategy (S1 = blue, S2 = green, S3 = red) and the missingness approach used (ALL, IMP-H, IMP-HC).

Mean estimates derived from models fitted at the level of total costs (S1, blue color) show a considerable degree of variability and slightly positive (ALL), slightly negative (IMP-HC), or quite negative (IMP-H) values. Similar conclusions can be drawn based on the results from models fitted at the level of the costs at each time (S2, green color). However, results derived from direct modeling of HRU data (S3, red color) show average estimates in favor of the intervention (slightly cheaper than the control) and located between the results under ALL and the other zero-imputation approaches (for S1 and S2). Although no formal bias assessment is possible, due to the empirical nature of the analyses, we note that for aggregated modeling strategies (S1 and S2), estimates based on some form of zero imputation (IMP-H, IMP-HC, IMP-H) are systematically lower compared with those based on models in which no ad hoc imputation was done (ALL). This suggests that reliance on the zero imputation of HRU/costs, which distorts the original data and likely leads to an underestimation of missingness uncertainty, can substantially drive the model estimates.

## Economic Evaluation

We end by assessing the cost-effectiveness of the intervention, comparing the results under each modeling strategy. We specifically rely on the examination of the cost-effectiveness plane (CEP)^37^ and the cost-effectiveness acceptability curve (CEAC)^38^ to summarize the health economic analysis.

**Figure 5:**
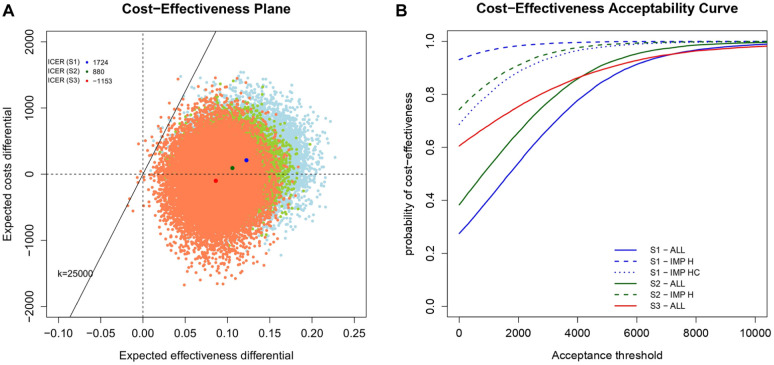
(a) Cost-effectiveness planes (CEPs) and (b) cost-effectiveness acceptability curves (CEACs) associated with alternative modeling strategies and missingness approaches. In the CEPs, incremental cost-effectiveness ratios based on the results from ALL under the 3 modeling strategies are indicated with corresponding darker colored dots (blue for S1, green for S2, and red for S3), while the straight line passing through the plot (evaluated at k=ϵ25,000 ) denotes the acceptance threshold value. In the CEACs, in addition to the results from ALL under each modeling strategy (solid lines), the probability values for the alternative missingness approaches (IMP-H and IMP-HC) are denoted with different types of blue (within S1) and green (within S2) dashed lines.

The CEP (Figure 5a) is a graphical representation of the joint distribution of the population average effectiveness and costs increments between the arms. In the graph, we show the results only under ALL (light blue for S1, light green for S2, and light red for S3) to ease presentation. The slope of the straight line crossing the plane is the willingness-to-pay threshold (often indicated as k ), with points lying below the line defining the sustainability area, where the treatment is considered more cost-effective than the control is. In the graph, we also show the incremental cost-effectiveness ratio (ICER) computed under each approach and denoted it with a darker colored dot. For all strategies, almost all samples fall in the North-East and South-East quadrants quite close to each other, although the clouds of dots are progressively shifted downward moving from S1 to S3, with the latter also displaying a slightly negative ICER. This suggests that, even though results obtained under the different modeling strategies do not largely differ, slightly more favorable conclusions for the intervention group are observed under S3.

The CEAC (Figure 5b) is obtained as the proportion of dots lying in the sustainability area upon varying the acceptance threshold k . For this analysis, based on current practice, we considered a range of k up to £10,000 per QALY gained. The CEAC displays the probability of cost-effectiveness, thus providing a simple summary of the uncertainty associated with the “optimal” decision making suggested by the ICER. Interestingly, results obtained under ALL (solid lines) for all strategies are associated with estimates that are systematically lower compared with zero-imputation strategies under S1 and S2, that is, either IMP-H (dashed lines) or IMP-HC (dotted line), for all k values. Finally, we note that results from different strategies without zero imputation (solid lines) show quite similar cost-effectiveness conclusions for k>£3,000 . This suggests how, across the scenarios explored and dataset used, differences in cost-effectiveness conclusions are mainly driven by the different approaches used to handle missing cases rather than the modeling strategies.

## Discussion

Trial-based economic evaluations are typically conducted on quantities that are derived from disaggregated data, such as self-reported EQ-5D and HRU questionnaires, which are almost inevitably affected by missingness. For HRU outcomes, the lack of a “gold standard” method of data collection^39^ and the typical occurrence of item nonresponse patterns make the task of handling missingness particularly challenging. Analysts routinely rely on some ad hoc methods to handle missing values that often involve specific assumptions, for example, zero-imputed corresponding to no use of service. Although this might be reasonable in some cases, zero imputations is often performed to ensure a higher completeness rate for more aggregated outcomes. This, however, can be a dangerous practice in that it fails to fully recognize the impact of missingness uncertainty and may even distort estimates and mislead cost-effectiveness assessments. Although this problem may be less relevant in some cases, for example, when disaggregated data are either fully observed or fully missing at each moment of collection (e.g., EQ-5D questionnaires), it becomes crucial when data are affected by item-level missingness, which is typically the case for HRU questionnaires.

In this article, we have presented a general modeling framework to handle item-level HRU missingness without requiring any ad hoc imputations. The framework takes advantage of the Bayesian setting to handle different features of the data while also directly quantifying the impact of missingness uncertainty on cost-effectiveness results. Our approach represents an improvement with respect to the current practice and can be implemented in a relatively easy way using freely available software.

In the PBS study, analyses implemented without zero imputation resulted in estimates of cost-effectiveness that were considerably lower compared with approaches based on zero-imputed data, especially for low values of the acceptance threshold (between 30%and50% lower for k<£4,000 ). These results suggest that model estimates can be highly affected by the approach used, with models fitted at more aggregated levels possibly requiring more restrictive assumptions about missing HRUs (e.g., assumed zeros) or the discard of partially observed cases, which may lead to underestimation of the impact of missingness uncertainty and a substantial loss of information.

According to these considerations and previous guidelines,^40^ we formulate the following recommendations for analysts. First, at the design stage, appropriate strategies should be used to minimize the number of missing values in the data collected during the study^41^ such as reducing the length of the follow-up period and/or the number of items within the questionnaires. Second, once the data are collected, the practice of ad hoc imputing HRU data (e.g., zero) should be avoided, unless clearly motivated, since it can distort the data and lead to incorrect inferences. Third, at the analysis stage, the choice of the modeling approach should be informed based on the observed missingness patterns. When individuals are associated with fully missing responses in questionnaires and over time points, no advantage can be obtained by focusing on disaggregated data, and modeling at the level of QALYs and total costs is likely to be appropriate. When individuals are associated with fully missing responses in questionnaires but partially observed responses across time points, a model fitted at the level of longitudinal utilities and costs should be preferred. When individuals are associated with partially missing responses within questionnaires and across time points, only a model fitted at the level of questionnaire responses (e.g., HRU categories) allows for full use of all the available evidence collected. Note that, in practice, the implementation of models at more disaggregated levels becomes more challenging as the number of outcomes, time points, and missing values increases. Thus, analysts should consider the feasibility of the ideal approach in relation to the available data and, when necessary, implement strategies to facilitate its implementation. Examples include simplification of the dependence structure among the modeled variables to reduce the number of parameters to estimate, the use of more informative priors to handle sparse data, and aggregation of some types of disaggregated data (e.g., different types of health care categories) to reduce the number of modeled variables.

Results obtained from a traditional approach, based on multiple imputation by chained equations (MICE) and bootstrapping (shown in Appendix A), lead to estimates and cost-effectiveness conclusions that were in line with those from Bayesian models. However, we encountered some practical issues that limited the implementation of the traditional approach. First, all attempts to apply the method at the level of HRU categories led to substantial convergence problems in the MICE algorithm. Second, on a standard computer with a 16 GB RAM and 4 cores, bootstrap methods substantially increased the computational time needed to run the analysis to about 4 h, compared with a maximum of 1 h required to fit any of the Bayesian models. We therefore conclude that, although traditional approaches can be used to perform the analysis, in practice their implementation is likely to become more challenging and computationally demanding compared with a Bayesian approach, especially when the complexity of the analysis model is increased to account for the features of the data (e.g., nonnormality, correlation) and to quantify the impact of different sources of uncertainty on the results (e.g., missing data, decision making).

Our results are obtained with specific reference to the case study considered. However, the PBS trial is very much representative of the “typical” dataset used in health economic evaluation alongside studies. Thus, it is highly likely that the same features (and potentially the same contradictions in the results, upon varying the complexity of the modeling assumptions) apply to many real cases. We note that the decision-theoretic framework granted by the Bayesian approach provides a natural setting to quantify the impact of the uncertainty on the results while also allowing an intuitive probabilistic interpretation of standard cost-effectiveness outputs (e.g., CEACs). For example, if results are not robust to a set of departures from the benchmark missingness assumption, further analyses can be sought by means of more advanced methods to explicitly investigate the variability in the unobserved values based on external information (e.g., selection models or pattern mixture models).^42,43^

There are 2 main limitations of the proposed framework. First, the choice of treating partially observed data for each variable under a common missingness assumption may be practically convenient but not realistic. It is plausible that the presence of different missingness patterns suggests the existence of different missingness processes, for example, people who drop out of the study are likely associated with different reasons with respect to those who missed a few visits, and such processes should be specified separately. Second, the model may become computationally challenging when the number of variables (i.e., time points or HRU categories) increases. Alternative approaches could be used to overcome these limitations. For example, the sparsity of the data could be handled by either aggregating some variables based on some plausible justifications (e.g., aggregating different types of GP visits as a single HRU category and computing a weighted price) or by using shared priors to use the information from the observed data across all or some of the variables to facilitate the identification of some parameters.

In conclusion, in this work we have presented a flexible Bayesian analytic framework that can 1) jointly model HRUs and effectiveness, 2) account for the features of the data, and 3) make full use of the available evidence to quantify the impact of missingness uncertainty without the need to rely on ad hoc imputations prior to model fitting. Unless clearly justified based on some external information, analyses that rely on these ad hoc imputations prior to model fitting will produce results that are likely driven by the specific approach used (and therefore implicit assumptions made) and should therefore be avoided.

## Supplemental Material

sj-pdf-1-mdm-10.1177_0272989X251376026 – Supplemental material for A Bayesian Modeling Framework for Health Care Resource Use and Costs in Trial-Based Economic Evaluations Supplemental material, sj-pdf-1-mdm-10.1177_0272989X251376026 for A Bayesian Modeling Framework for Health Care Resource Use and Costs in Trial-Based Economic Evaluations by Andrea Gabrio in Medical Decision Making

## Figures and Tables

**Figure 6:**
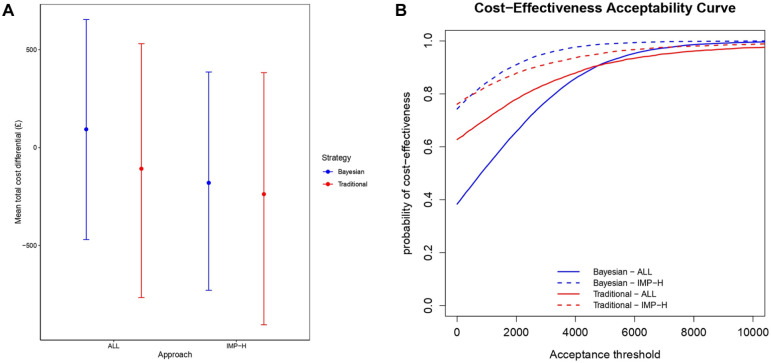
Estimated mean total cost difference with related 95% credible/confidence interval (Panel A) and CEAC (Panel B) obtained based on a traditional (red dots and lines) or Bayesian (blue dots and lines) analysis, under alternative missingness approaches: all data with no zero-imputation (ALL - solid lines) or with zero-imputation (IMP-H - dotted lines).

## Appendix A: Estimates from the Models Fitted at Different Levels

### A.1 Model at the Level of Total Costs

Table 4 shows the posterior estimates and 95% credible intervals for the marginal mean total costs and QALYs by treatment group ( $\mu_{\mathrm{tc}},\mu_{e}$ ). Estimates are obtained based on 3 different strategies to handle partially observed HRU/cost data prior to fitting the model at the level of total costs and QALYs. These include all data with no zero imputation of partially observed HRU or cost cases (ALL), zero imputation of partially observed HRU cases and no zero imputation for cost cases (IMP-H), and zero imputation of partially observed HRU and cost cases (IMP-HC).

**Table 4:** Posterior Means and 95% Credible Intervals for $\mu_{\mathrm{tc}}$ and $\mu_{e}$ in the Control ( t=1 ) and Intervention ( t=2 ) under 3 Approaches to Handle Partially Observed Cases When Fitting the Model at the Level of Total Costs and QALYs

Approach	Outcome	$\mu_{\left(t=1\right)}$		$\mu_{\left(t=2\right)}$	
		Mean	95% CI	Mean	95% CI
ALL	Total cost	2,543	(2,157; 2,938)	2,754	(2,249; 3,310)
	QALY	0.487	(0.449; 0.575)	0.609	(0.569; 0.649)
IMP-H	Total cost	2,888	(2,411; 3,393)	2,379	(1,939; 2,872)
	QALY	0.488	(0.45; 0.526)	0.61	(0.571; 0.651)
IMP-HC	Total cost	2,395	(1,897; 2,899)	2,237	(1,175; 2,749)
	QALY	0.486	(0.449; 0.523)	0.61	(0.572; 0.649)

QALY, quality-adjusted life-year.

### A.2 Model at the Level of Costs

Table 5 shows the posterior estimates and 95% credible intervals for the marginal mean costs and utilities at each follow-up by treatment group ( $\mu_{\mathrm{cj}},\mu_{\mathrm{uj}}$ ). Estimates are obtained based on 2 different strategies to handle partially observed HRU/cost data prior to fitting the model at the level of the costs and utilities. These include all data with no zero imputation of partially observed HRU or cost cases (ALL) and zero imputation of partially observed HRU cases (IMP-H).

**Table 5:** Posterior Means and 95% Credible Intervals for $\mu_{\mathrm{cj}}$ and $\mu_{\mathrm{uj}}$ in the Xontrol ( t=1 ) and Intervention ( t=2 ) under 2 Approaches to Handle Partially Observed Cases When Fitting the Model at the Level of the Costs and Utilities

Approach	Outcome	*μ*(*t* = 1)				μ(t=2)			
		j=1		*j* = 2		j=1		j=2	
		Mean	95% CI	Mean	95% CI	Mean	95% CI	Mean	95% CI
ALL	Cost	1,311	(1,085; 1,549)	1,296	(1,041; 1,588)	1,443	(1,142; 1,770)	1,257	(985; 1,545)
	Utility	0.491	(0.438; 0.544)	0.633	(0.578; 0.693)	0.480	(0.432; 0.529)	0.619	(0.567; 0.672)
IMP-H	Cost	1,187	(996; 1,446)	1,265	(1,006; 1,546)	1,118	(858; 1,394)	1,154	(885; 1,451)
	Utility	0.491	(0.439; 0.545)	0.633	(0.575; 0.69)	0.480	(0.431; 0.527)	0.619	(0.565; 0.67)

### A.3 Model at the Level of HRUs

Table 6 shows the posterior estimates and 95% credible intervals for the marginal HRU rates for each resource use category k and the marginal utility means at each follow-up by treatment group ( $\mu_{\mathrm{hruj}}^{k},\mu_{\mathrm{uj}}$ ). Estimates are obtained based on a model fitted at the HRU and utility level using all data without any zero imputation of partially observed cases (ALL).

**Table 6:** Posterior Means and 95% Credible Intervals for $\mu_{\mathrm{hruj}}^{k}$ and $\mu_{\mathrm{uj}}$ in the Control ( t=1 ) and Intervention ( t=2 ) without Prior Data Imputation When Fitting the Model at the Level of the Health Care Resource Use and Utility

Outcome	μ(t=1)				μ(t=2)			
	j=1		j=2		j=1		j=2	
	Mean	95% CI	Mean	95% CI	Mean	95% CI	Mean	95% CI
PSYDR	1.36	(1.18; 1.56)	1.38	(1.19; 1.58)	1.23	(1.04; 1.16)	0.97	(0.8; 1.16)
PSYCH	0.4	(0.29; 0.51)	0.71	(0.56; 0.86)	0.33	(0.22; 0.44)	0.27	(0.18; 0.37)
PHYSI	0.7	(0.56; 0.85)	0.18	(0.11; 0.25)	0.43	(0.24; 0.47)	0.35	(0.24; 0.47)
DENT	1.05	(0.87; 1.23)	0.9	(0.74; 1.06)	1.08	(0.87; 1.09)	1	(0.8; 1.2)
SOCWORK	1.07	(0.91; 1.26)	1.08	(0.91; 1.26)	0.88	(0.71; 1.04)	1.34	(1.12; 1.57)
COMWORK	3.83	(3.44; 4.21)	10.18	(9.43; 10.97)	7.18	(6.08; 8.36)	4.35	(3.73; 4.99)
GP	7.86	(7.19; 8.55)	6.63	(3.4; 12.1)	4.61	(4.18; 5.05)	4.59	(4.16; 5.02)
NURSE	8.83	(8.32; 9.33)	2.02	(1.78; 2.27)	3.12	(2.79; 3.48)	3.37	(3.01; 3.72)
THERAP	5.43	(5.03; 5.83)	3.98	(3.63; 4.31)	6.85	(6.35; 7.37)	6.6	(6.41; 7.11)
Utility	0.534	(0.469; 0.6)	0.632	(0.574; 0.693)	0.483	(0.426; 0.54)	0.617	(0.57; 0.663)

### A.4 Traditional Approach

Table 7 shows the estimates and 95% bootstrap confidence intervals (computed using the percentile method) for the marginal mean total costs and QALYs by treatment group ( $\mu_{\mathrm{tc}},\mu_{e}$ ). Estimates are obtained based on the traditional approach, which relies on the following methods: missing utility and cost data at each time are first imputed 20 times (under Missing At Random - MAR) using MICE and a predictive mean matching imputation method; total cost and QALYs are then computed using imputed cost and utility data; seemingly unrelated regression models are then fitted to each imputed dataset, and Rubin’s rules are used to pool estimates of $\mu_{\mathrm{tc}}$ and $\mu_{e}$ across imputed datasets. Finally, the process is embedded within a nonparametric bootstrap procedure with 5,000 bootstrap samples to empirically approximate the sampling distribution of $\mu_{\mathrm{tc}}$ and $\mu_{e}$ . To ease the comparison with the results obtained under the Bayesian models from section A.2, estimates are reported under 2 alternative strategies for dealing with partially observed HRU/cost data prior to model fitting: all data with no imputation of missing HRU or cost cases (ALL) and zero imputation of missing HRU cases (IMP-H).

**Table 7:** Bootstrapped Means and 95% Confidence Intervals (Computed Using the Percentile Method) for $\mu_{\mathrm{tc}}$ and $\mu_{e}$ in the Control ( t=1 ) and Intervention ( t=2 ) Obtained under the Traditional Analysis and Based on 2 Alternative Approaches to Handle Partially Observed Cases (ALL and IMP-H)

Approach	Outcome	$\mu_{\left(t=1\right)}$		$\mu_{\left(t=2\right)}$	
		Mean	95% CI	Mean	95% CI
ALL	Total cost	2,473	(1,969; 3,043)	2,366	(1,817; 2,968)
	QALY	0.505	(0.461; 0.548)	0.587	(0.539; 0.636)
IMP-H	Total cost	2,395	(1,874; 2,950)	2157	(1,651; 2,674)
	QALY	0.504	(0.461; 0.547)	0.587	(0.539; 0.634)

QALY, quality-adjusted life-year.

Figure 6 compares the estimated mean total cost differences (panel a) and the CEACs (panel b) obtained from a Bayesian and traditional analysis fitted at the level of utility/cost data from the PBS study, either under the ALL or IMP-H missingness approach.

## Appendix B: Implementation “Trick” to Handle Zero Values

The model specified at the most disaggregated level of the data uses a different sampling distribution for the k -th HRU category, depending on the observed value of the indicator $d_{\mathrm{ihru}}^{k}$

$${\mathrm{hru}}_{i}^{k}|d_{\mathrm{ihru}}\sim \left\{ \begin{array}{c} p\left(\mathrm{hr}u_{i}|d_{\mathrm{ihru}}=0\right)=p\left(\mathrm{hr}u_{i}|\theta^{>0}\right),\mathrm{if}\;\mathrm{hr}u_{i}>0\\ p\left(\mathrm{hr}u_{i}|d_{\mathrm{ihru}}=1\right)=p\left(\mathrm{hr}u_{i}|\theta^{0}\right),\;\mathrm{if}\;\mathrm{hr}u_{i}=0, \end{array}\right.$$

where $\theta^{>0}$ and $\theta^{0}$ denote the sets of all parameters indexing the model fitted to the positive and structural zero components of the HRU data. The model for ${\mathrm{hru}}_{i}^{k}=1$ is degenerate at a point mass at 0, while that for ${\mathrm{hru}}_{i}^{k}>0$ is defined in terms of a standard probability distribution, for example, normal. We can conveniently rewrite this more succinctly and with specific reference to our case as

$${\mathrm{hru}}_{i}^{k}\sim \mathrm{Normal}\left(\phi_{\mathrm{ihru}}^{d_{\mathrm{ihru}}},\psi_{\mathrm{ihru}}^{d_{\mathrm{ihru}}}\right).$$

If we set the mean of the structural-zero component $\phi_{\mathrm{ihru}}^{0}=0$ and select $\psi_{\mathrm{ihru}}^{0}$ equal to some value to induce a variance as close to 0 as possible, the 2 specifications are identical. More specifically, the required behavior is very closely mimicked if we define our model with

$$\phi_{\mathrm{ihru}}^{0}=\alpha_{0}\;[+\ldots ]$$

and set $\alpha_{0}=0$ and $\sigma_{\mathrm{hru}}\approx 0$ , which implies setting the mean of the structural-zero component equal to 0 with virtually no uncertainty. In other words, we can specify extremely informative priors on the parameters $\theta^{0}$ so that the implied distribution for the structural-zero components of the mixture is concentrated around 0 with essentially no uncertainty. More importantly, with such a prior, no amount of data can modify the posterior. The critical aspect of this strategy, however, is that inferences may be potentially sensitive to the way such priors are specified, that is, whether a small variation in the hyperprior values can affect the posterior estimates.

In fact, the estimation of the other parameters is not really affected by this choice, provided that the encoded prior really induces the variance toward zero. It is also plausible that different values for $\sigma_{\mathrm{hru}}^{0}$ have an impact on measures of model fit, such as the deviance information criterion (DIC). This is essentially due to the fact that the population is really composed of 2 groups, one of which shows HRUs that are identically zero. Thus, the closer the approximation to zero for the variance, the better the fit to the observed data and therefore the smaller the resulting DIC.

With this in mind, we have used different values for $\sigma_{\mathrm{hru}}^{0}$ to assess the impact on the mean HRU estimates. We have explored a range of possibilities by progressively decreasing the value of this parameter (e.g., between 0.001 and 0.000001 ) and assessed their impact on posterior results. Results in terms of mean posterior estimates and 95% credible intervals were almost unchanged in all cases. Thus, we can assert that model performance was unaffected by the choice of the value for $\sigma_{\mathrm{hru}}^{0}$ . We also observe that the DIC becomes smaller when the standard deviation parameter decreases and the best-fitting model is the one associated with the smallest values, although the results are hardly different from both an estimation and convergence perspective for all the parameters.
