# Supplementary material for: A Bayesian Modeling Framework for Health Care Resource Use and Costs in Trial-Based Economic Evaluations
Source: Med Decis Making. 2025 Oct 23;46(2):158–73. doi: 10.1177/0272989X251376026 (PMC12769925; doi:10.1177/0272989X251376026)
Supplement: sj-pdf-1-mdm-10.1177_0272989X251376026 – Supplemental material for A Bayesian Modeling Framework for Health Care Resource Use and Costs in Trial-Based Economic Evaluations [file sj-pdf-1-mdm-10.1177_0272989X251376026.pdf]

Supplementary Material for: A modelling framework for health care resource use and costs in trial-based economic evaluations

A Empirical Distributions of HRU Variables

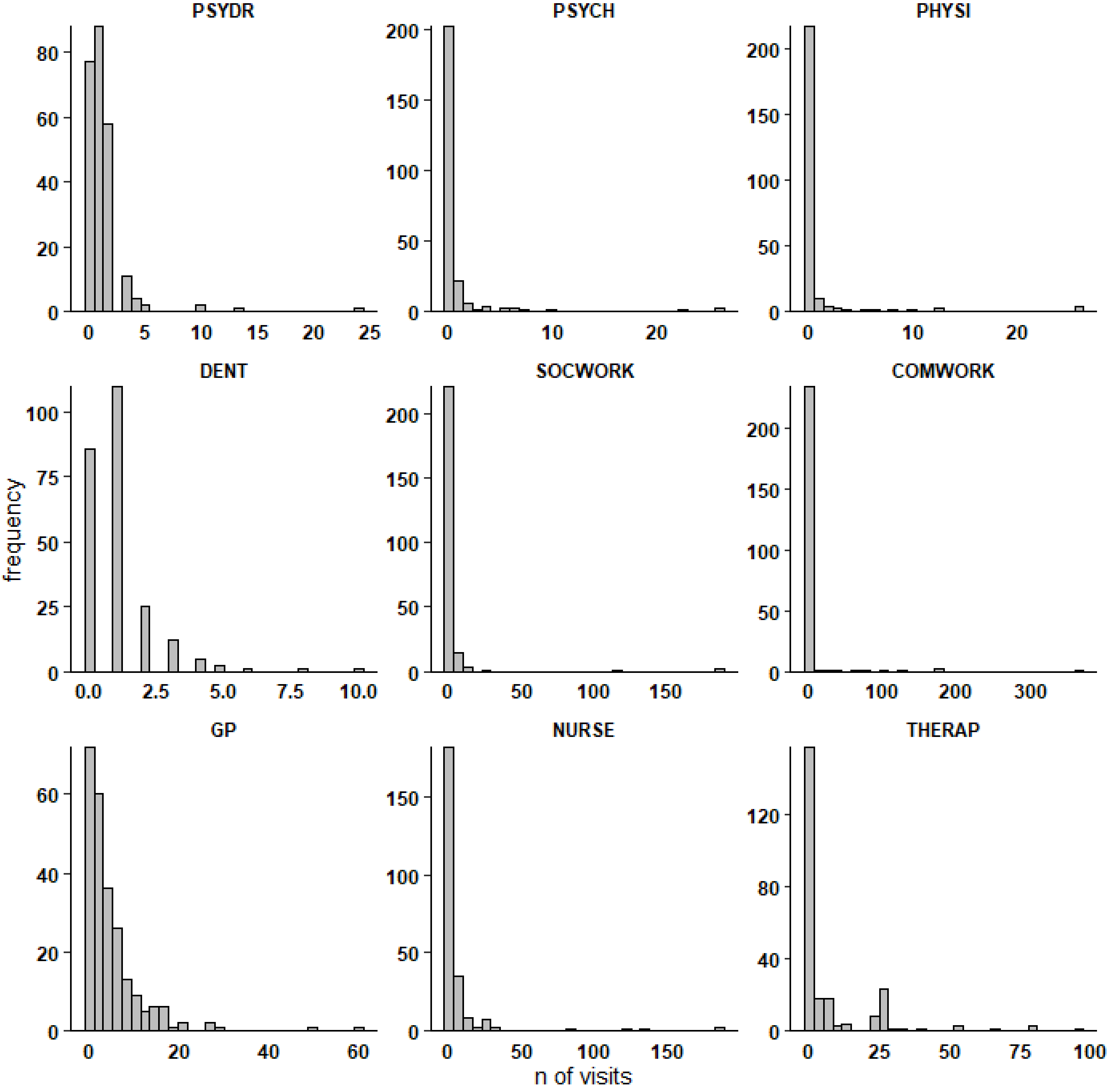

Figure 1. Empirical distributions based on the number of HRU visits collected at baseline for each participant in the PBS study for nine different types healthcare services: psychiatric doctor (PSYDR), psychologist (PSYCH), physiotherapist (PHYSI), dentist (DENT), social worker (SOCWORK). community worker (COMWORK), general practitioner (GP), nurse (NURSE), alternative therapist (THERAP). Due to the different ranges for each HRU variable, histograms are plotted on different scales for illustrative purposes.

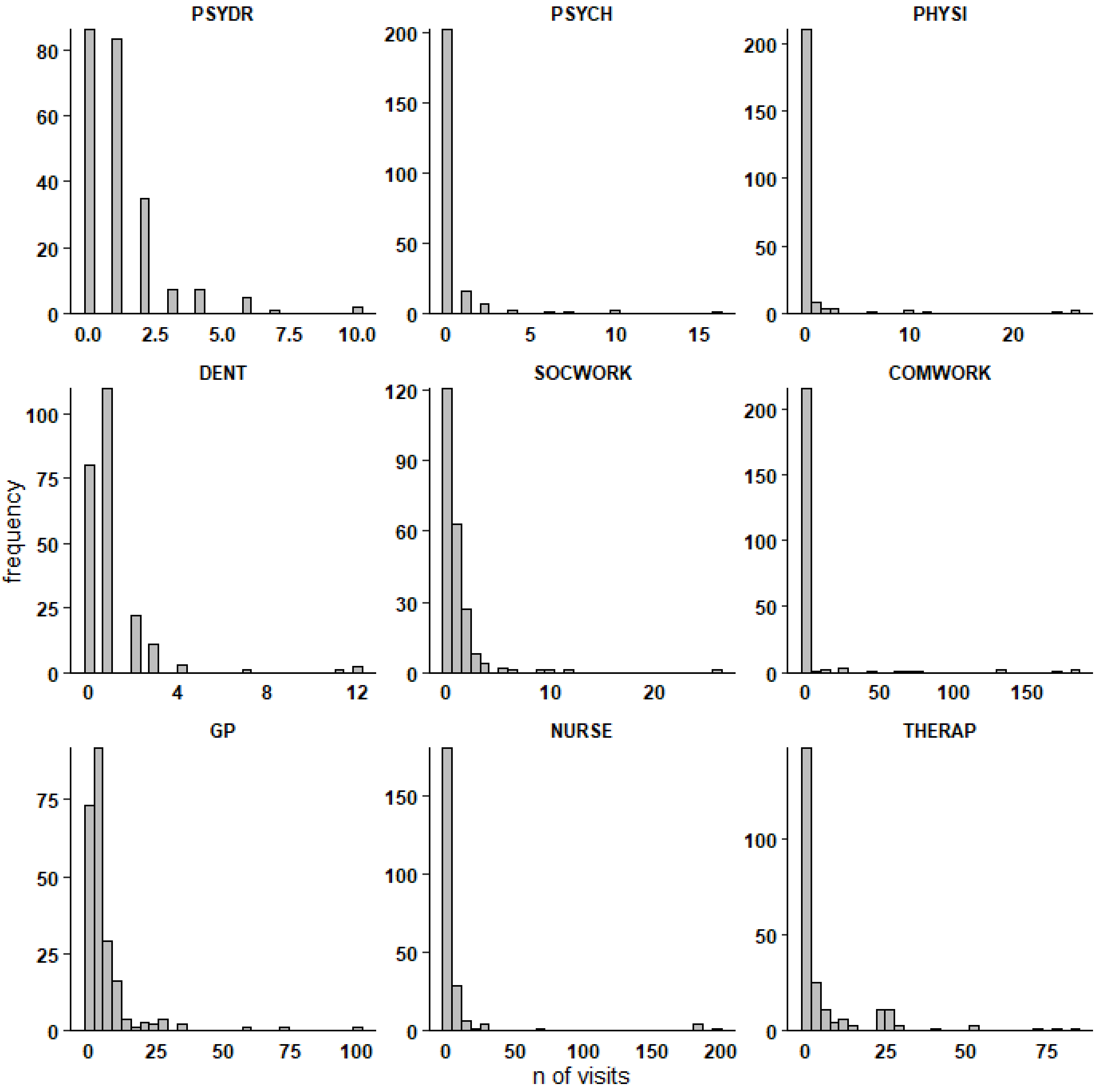

Figure 2. Empirical distributions based on the number of HRU visits collected at 6 months follow-up for each participant in the PBS study for nine different types healthcare services: psychiatric doctor (PSYDR), psychologist (PSYCH), physiotherapist (PHYSI), dentist (DENT), social worker (SOCWORK). community worker (COMWORK), general practitioner (GP), nurse (NURSE), alternative therapist (THERAP). Due to the different ranges for each HRU variable, histograms are plotted on different scales for illustrative purposes.

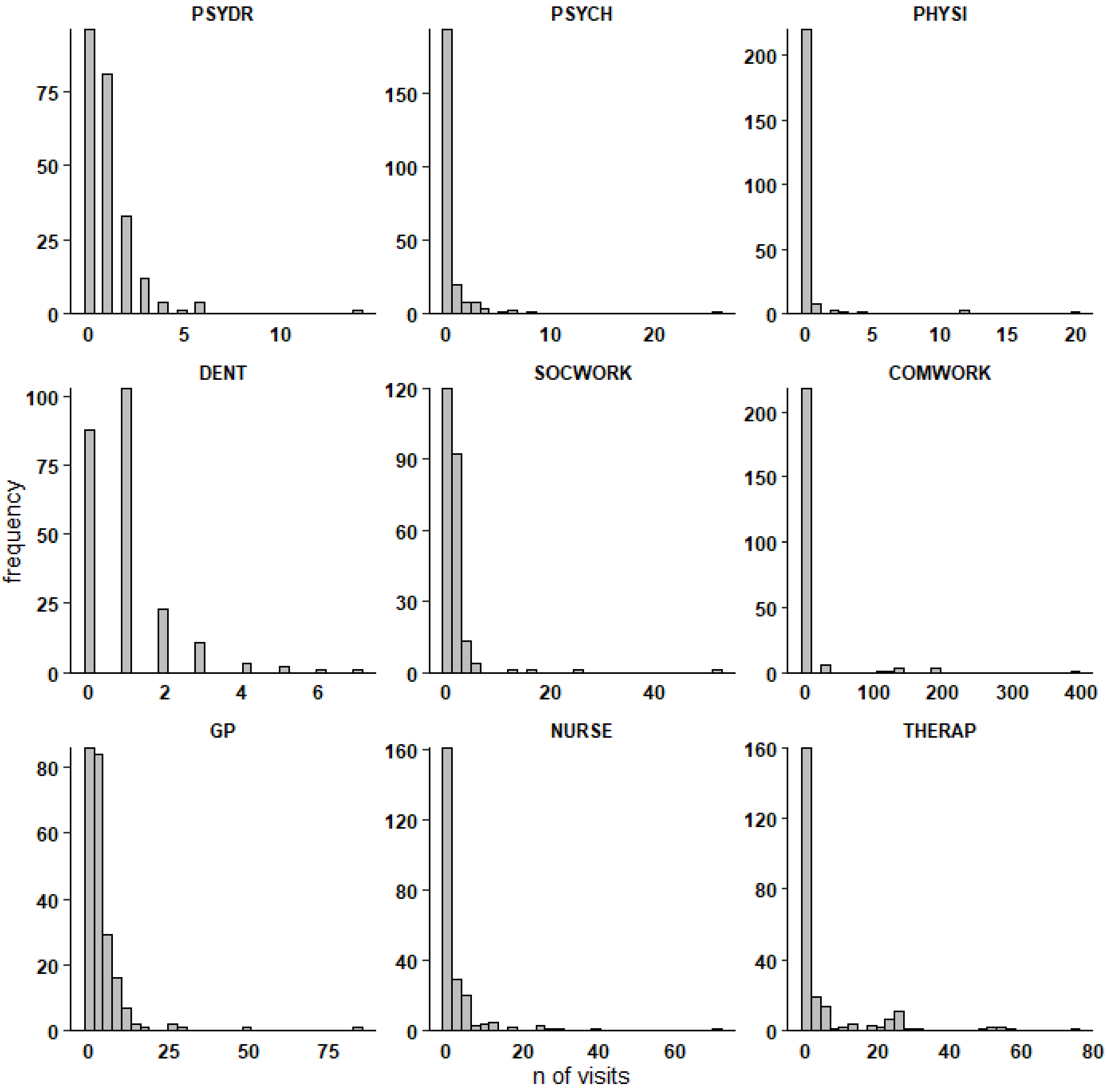

Figure 3. Empirical distributions based on the number of HRU visits collected at 12 months follow-up for each participant in the PBS study for nine different types healthcare services: psychiatric doctor (PSYDR), psychologist (PSYCH), physiotherapist (PHYSI), dentist (DENT), social worker (SOCWORK). community worker (COMWORK), general practitioner (GP), nurse (NURSE), alternative therapist (THERAP). Due to the different ranges for each HRU variable, histograms are plotted on different scales for illustrative purposes.

B JAGS model code

B.1 Model code for total costs and QALYs

```
model{

# the likelihood function for dependent variables
for (i in 1:N) {
tau_TC[i] <- mu_TC[i]/pow(sd_TC[group[i]+1], 2)
log(mu_TC[i]) <- alpha0 + alpha1*group[i] + alpha2*(TC_0[i] - mean(TC_0[]))
AG_TC[i] ~dgamma(mu_TC[i]*tau_TC[i], tau_TC[i])

mu_QALY[i] <- beta0_QALY + beta1_QALY*group[i] + beta2_QALY*(EQ5D0[i] - mean(EQ5D0[])) +
beta3_QALY*(AG_TC[i] - mean(AG_TC[]))
QALY[i] ~dnorm(mu_QALY[i], tau_QALY)

mu_EQ5D0[i] <- beta0_EQ5D0 + beta1_EQ5D0*(TC_0[i] - mean(TC_0[]))
EQ5D0[i] ~dnorm(mu_EQ5D0[i], tau_EQ5D0)

}

# priors on mean and std

tau_EQ5D0 <- pow(sd_EQ5D0, -2)
tau_QALY <- pow(sd_QALY, -2)

for(g in 1:2){
sd_TC[g] ~dunif(0, 100)
}

sd_EQ5D0 ~dunif(0, 100)
sd_QALY ~dunif(0, 100)

# priors on level regression coefficients
alpha0 ~dnorm (0, 0.01)
alpha1 ~dnorm (0, 0.01)
alpha2 ~dnorm (0, 0.01)
beta0_QALY ~dnorm(0,0.01)
beta1_QALY ~dnorm(0,0.01)
beta2_QALY ~dnorm(0,0.01)
beta3_QALY ~dnorm(0,0.01)
beta0_EQ5D0 ~dnorm(0,0.01)
beta1_EQ5D0 ~dnorm(0,0.01)

} # end of the model block
```

B.2 Model code for costs and utilities

```
model{

# the likelihood function for dependent variables
for (i in 1:N) {
tau_TC1[i] <- mu_TC1[i]/pow(sd_TC1[group[i]+1], 2)
log(mu_TC1[i]) <- alpha0[1] + alpha1[1]*group[i] + alpha2[1]*(TC_0[i] - mean(TC_0[])) +
alpha3[1]*(EQ5D0[i] - mean(EQ5D0[]))
TC_1[i] ~dgamma(mu_TC1[i]*tau_TC1[i], tau_TC1[i])
```

```
tau_TC2[i] <- mu_TC2[i]/pow(sd_TC2[group[i]+1], 2)
log(mu_TC2[i]) <- alpha0[2] + alpha1[2]*group[i] + alpha2[2]*(TC_1[i] - mean(TC_1[])) +
alpha3[2]*(EQ5D1[i] - mean(EQ5D1[]))
TC_2[i] ~dgamma(mu_TC2[i]*tau_TC2[i], tau_TC2[i])

TC_0[i] ~dnorm(mu_TC0*tau_TC0, tau_TC0)

mu_EQ5D2[i] <- beta0_EQ5D2 + beta1_EQ5D2*group[i] + beta2_EQ5D2*(TC_2[i] - mean(TC_2[])) +
beta3_EQ5D2*(EQ5D1[i] - mean(EQ5D1[]))
EQ5D2[i] ~dnorm(mu_EQ5D2[i], tau_EQ5D2)

mu_EQ5D1[i] <- beta0_EQ5D1 + beta1_EQ5D1*group[i] + beta2_EQ5D1*(TC_1[i] - mean(TC_1[])) +
beta3_EQ5D1*(EQ5D0[i] - mean(EQ5D0[]))
EQ5D1[i] ~dnorm(mu_EQ5D1[i], tau_EQ5D1)

mu_EQ5D0[i] <- beta0_EQ5D0 + beta1_EQ5D0*(TC_0[i] - mean(TC_0[]))
EQ5D0[i] ~dnorm(mu_EQ5D0[i], tau_EQ5D0)
}

# parameter back-transformations

tau_TC0 <- mu_TC0/pow(sd_TC0, 2)
tau_EQ5D0 <- pow(sd_EQ5D0, -2)
tau_EQ5D1 <- pow(sd_EQ5D1, -2)
tau_EQ5D2 <- pow(sd_EQ5D2, -2)

# priors on mean and std

mu_TC0 ~dunif(0, 100)
sd_TC0 ~dunif(0, 100)
sd_EQ5D0 ~dunif(0, 100)
sd_EQ5D1 ~dunif(0, 100)
sd_EQ5D2 ~dunif(0, 100)

for(g in 1:2){
sd_TC1[g] ~dunif(0, 100)
sd_TC2[g] ~dunif(0, 100)
}

# priors on level regression coefficients
for(l in 1:2){
alpha0[l] ~dnorm (0, 0.01)
alpha1[l] ~dnorm (0, 0.01)
alpha2[l] ~dnorm (0, 0.01)
alpha3[l] ~dnorm (0, 0.01)
}

beta0_EQ5D0 ~dnorm(0,0.01)
beta1_EQ5D0 ~dnorm(0,0.01)
beta0_EQ5D1 ~dnorm(0,0.01)
beta1_EQ5D1 ~dnorm(0,0.01)
beta2_EQ5D1 ~dnorm(0,0.01)
beta3_EQ5D1 ~dnorm(0,0.01)
beta0_EQ5D2 ~dnorm(0,0.01)
beta1_EQ5D2 ~dnorm(0,0.01)
beta2_EQ5D2 ~dnorm(0,0.01)
beta3_EQ5D2 ~dnorm(0,0.01)

} # end of the model block
```

B.3 Model code for HRUs and utilities

```
model{

# the likelihood function for dependent variables
for (i in 1:N) {
#models for 9 HRU outcome types at 3 times

mu_EQ5D1[i] <- beta0_EQ5D[1] + beta1_EQ5D[1]*group[i] + beta2_EQ5D[1]*(EQ5D0[i] - mean(EQ5D0[])) +
beta_EQ5D1[1]*(HRU_PSYDR1[i] - mean(HRU_PSYDR1[])) +
beta_EQ5D1[2]*(HRU_PSYCH1[i] - mean(HRU_PSYCH1[])) +
beta_EQ5D1[3]*(HRU_PHYSI1[i] - mean(HRU_PHYSI1[])) +
beta_EQ5D1[4]*(HRU_DENT1[i] - mean(HRU_DENT1[])) +
beta_EQ5D1[5]*(HRU_SOCWORK1[i] - mean(HRU_SOCWORK1[])) +
beta_EQ5D1[6]*(HRU_COMWORK1[i] - mean(HRU_COMWORK1[])) +
beta_EQ5D1[7]*(HRU_GP1[i] - mean(HRU_GP1[])) +
beta_EQ5D1[8]*(HRU_NURSE1[i] - mean(HRU_NURSE1[])) +
beta_EQ5D1[9]*(HRU_THERAP1[i] - mean(HRU_THERAP1[]))
EQ5D1[i] ~dnorm(mu_EQ5D1[i], tau_EQ5D1[group[i]+1])
mu_EQ5D2[i] <- beta0_EQ5D[2] + beta1_EQ5D[2]*group[i] +
beta2_EQ5D[2]*(EQ5D1[i] - mean(EQ5D1[])) +
beta_EQ5D2[1]*(HRU_PSYDR2[i] - mean(HRU_PSYDR2[])) +
beta_EQ5D2[2]*(HRU_PSYCH2[i] - mean(HRU_PSYCH2[])) +
beta_EQ5D2[3]*(HRU_PHYSI2[i] - mean(HRU_PHYSI2[])) +
beta_EQ5D2[4]*(HRU_DENT2[i] - mean(HRU_DENT2[])) +
beta_EQ5D2[5]*(HRU_SOCWORK2[i] - mean(HRU_SOCWORK2[])) +
beta_EQ5D2[6]*(HRU_COMWORK2[i] - mean(HRU_COMWORK2[])) +
beta_EQ5D2[7]*(HRU_GP2[i] - mean(HRU_GP2[])) +
beta_EQ5D2[8]*(HRU_NURSE2[i] - mean(HRU_NURSE2[])) +
beta_EQ5D2[9]*(HRU_THERAP2[i] - mean(HRU_THERAP2[]))
EQ5D2[i] ~dnorm(mu_EQ5D2[i], tau_EQ5D2[group[i]+1])
mu_EQ5D0[i] <- beta0_EQ5D[3] + beta_EQ5D0[1]*(HRU_PSYDR0[i] - mean(HRU_PSYDR0[])) +
beta_EQ5D0[2]*(HRU_PSYCH0[i] - mean(HRU_PSYCH0[])) +
beta_EQ5D0[3]*(HRU_PHYSI0[i] - mean(HRU_PHYSI0[])) +
beta_EQ5D0[4]*(HRU_DENT0[i] - mean(HRU_DENT0[])) +
beta_EQ5D0[5]*(HRU_SOCWORK0[i] - mean(HRU_SOCWORK0[])) +
beta_EQ5D0[6]*(HRU_COMWORK0[i] - mean(HRU_COMWORK0[])) +
beta_EQ5D0[7]*(HRU_GP0[i] - mean(HRU_GP0[])) +
beta_EQ5D0[8]*(HRU_NURSE0[i] - mean(HRU_NURSE0[])) +
beta_EQ5D0[9]*(HRU_THERAP0[i] - mean(HRU_THERAP0[]))
EQ5D0[i] ~dnorm(mu_EQ5D0[i], tau_EQ5D0)

mu_PSYDR1[i] <- alpha0_PSYDR[1,S0_PSYDR[i]+1] + alpha1_PSYDR[1,S0_PSYDR[i]+1]*group[i] +
alpha2_PSYDR[1,S0_PSYDR[i]+1]*(HRU_PSYDR0[i] - mean(HRU_PSYDR0[])) +
alpha3_PSYDR[1,S0_PSYDR[i]+1]*(EQ5D0[i] - mean(EQ5D0[]))
HRU_PSYDR1[i] ~dnorm(mu_PSYDR1[i], tau_PSYDR1[group[i]+1,S0_PSYDR[i]+1])
mu_PSYDR2[i] <- alpha0_PSYDR[2,S0_PSYDR[i]+1] + alpha1_PSYDR[2,S0_PSYDR[i]+1]*group[i] +
alpha2_PSYDR[2,S0_PSYDR[i]+1]*(HRU_PSYDR1[i] - mean(HRU_PSYDR1[])) +
alpha3_PSYDR[2,S0_PSYDR[i]+1]*(EQ5D1[i] - mean(EQ5D1[]))
HRU_PSYDR2[i] ~dnorm(mu_PSYDR2[i], tau_PSYDR2[group[i]+1,S0_PSYDR[i]+1])
mu_PSYDR0[i] <- alpha0_PSYDR[3,S0_PSYDR[i]+1]
HRU_PSYDR0[i] ~dnorm(mu_PSYDR0[S0_PSYDR[i]+1], tau_PSYDR0[S0_PSYDR[i]+1])

mu_PSYCH1[i] <- alpha0_PSYCH[1,S0_PSYCH[i]+1] + alpha1_PSYCH[1,S0_PSYCH[i]+1]*group[i] +
alpha2_PSYCH[1,S0_PSYCH[i]+1]*(HRU_PSYCH0[i] - mean(HRU_PSYCH0[])) +
alpha3_PSYCH[1,S0_PSYCH[i]+1]*(EQ5D0[i] - mean(EQ5D0[]))
HRU_PSYCH1[i] ~dnorm(mu_PSYCH1[i], tau_PSYCH1[group[i]+1,S0_PSYCH[i]+1])
mu_PSYCH2[i] <- alpha0_PSYCH[2,S0_PSYCH[i]+1] + alpha1_PSYCH[2,S0_PSYCH[i]+1]*group[i] +
alpha2_PSYCH[2,S0_PSYCH[i]+1]*(HRU_PSYCH1[i] - mean(HRU_PSYCH1[])) +
alpha3_PSYCH[2,S0_PSYCH[i]+1]*(EQ5D1[i] - mean(EQ5D1[]))
HRU_PSYCH2[i] ~dnorm(mu_PSYCH2[i], tau_PSYCH2[group[i]+1,S0_PSYCH[i]+1])
mu_PSYCH0[i] <- alpha0_PSYCH[3,S0_PSYCH[i]+1]
```

```
HRU_PSYCH0[i] ~dnorm(mu_PSYCH0[S0_PSYCH[i]+1], tau_PSYCH0[S0_PSYCH[i]+1])

mu_PHYSI1[i] <- alpha0_PHYSI[1,S0_PHYSI[i]+1] + alpha1_PHYSI[1,S0_PHYSI[i]+1]*group[i] +
alpha2_PHYSI[1,S0_PHYSI[i]+1]*(HRU_PHYSI0[i] - mean(HRU_PHYSI0[])) +
alpha3_PHYSI[1,S0_PHYSI[i]+1]*(EQ5D0[i] - mean(EQ5D0[]))
HRU_PHYSI1[i] ~dnorm(mu_PHYSI1[i], tau_PHYSI1[group[i]+1,S0_PHYSI[i]+1])
mu_PHYSI2[i] <- alpha0_PHYSI[2,S0_PHYSI[i]+1] + alpha1_PHYSI[2,S0_PHYSI[i]+1]*group[i] +
alpha2_PHYSI[2,S0_PHYSI[i]+1]*(HRU_PHYSI1[i] - mean(HRU_PHYSI1[])) +
alpha3_PHYSI[2,S0_PHYSI[i]+1]*(EQ5D1[i] - mean(EQ5D1[]))
HRU_PHYSI2[i] ~dnorm(mu_PHYSI2[i], tau_PHYSI2[group[i]+1,S0_PHYSI[i]+1])
mu_PHYSI0[i] <- alpha0_PHYSI[3,S0_PHYSI[i]+1]
HRU_PHYSI0[i] ~dnorm(mu_PHYSI0[S0_PHYSI[i]+1], tau_PHYSI0[S0_PHYSI[i]+1])

mu_DENT1[i] <- alpha0_DENT[1,S0_DENT[i]+1] + alpha1_DENT[1,S0_DENT[i]+1]*group[i] +
alpha2_DENT[1,S0_DENT[i]+1]*(HRU_DENT0[i] - mean(HRU_DENT0[])) +
alpha3_DENT[1,S0_DENT[i]+1]*(EQ5D0[i] - mean(EQ5D0[]))
HRU_DENT1[i] ~dnorm(mu_DENT1[i], tau_DENT1[group[i]+1,S0_DENT[i]+1])
mu_DENT2[i] <- alpha0_DENT[2,S0_DENT[i]+1] + alpha1_DENT[2,S0_DENT[i]+1]*group[i] +
alpha2_DENT[2,S0_DENT[i]+1]*(HRU_DENT1[i] - mean(HRU_DENT1[])) +
alpha3_DENT[2,S0_DENT[i]+1]*(EQ5D1[i] - mean(EQ5D1[]))
HRU_DENT2[i] ~dnorm(mu_DENT2[i], tau_DENT2[group[i]+1,S0_DENT[i]+1])
mu_DENT0[i] <- alpha0_DENT[3,S0_DENT[i]+1]
HRU_DENT0[i] ~dnorm(mu_DENT0[S0_DENT[i]+1], tau_DENT0[S0_DENT[i]+1])

mu_SOCWORK1[i] <- alpha0_SOCWORK[1,S0_SOCWORK[i]+1] + alpha1_SOCWORK[1,S0_SOCWORK[i]+1]*group[i] +
alpha2_SOCWORK[1,S0_SOCWORK[i]+1]*(HRU_SOCWORK0[i] - mean(HRU_SOCWORK0[])) +
alpha3_SOCWORK[1,S0_SOCWORK[i]+1]*(EQ5D0[i] - mean(EQ5D0[]))
HRU_SOCWORK1[i] ~dnorm(mu_SOCWORK1[i], tau_SOCWORK1[group[i]+1,S0_SOCWORK[i]+1])
mu_SOCWORK2[i] <- alpha0_SOCWORK[2,S0_SOCWORK[i]+1] + alpha1_SOCWORK[2,S0_SOCWORK[i]+1]*group[i] +
alpha2_SOCWORK[2,S0_SOCWORK[i]+1]*(HRU_SOCWORK1[i] - mean(HRU_SOCWORK1[])) +
alpha3_SOCWORK[2,S0_SOCWORK[i]+1]*(EQ5D1[i] - mean(EQ5D1[]))
HRU_SOCWORK2[i] ~dnorm(mu_SOCWORK2[i], tau_SOCWORK2[group[i]+1,S0_SOCWORK[i]+1])
mu_SOCWORK0[i] <- alpha0_SOCWORK[3,S0_SOCWORK[i]+1]
HRU_SOCWORK0[i] ~dnorm(mu_SOCWORK0[S0_SOCWORK[i]+1], tau_SOCWORK0[S0_SOCWORK[i]+1])

mu_COMWORK1[i] <- alpha0_COMWORK[1,S0_COMWORK[i]+1] + alpha1_COMWORK[1,S0_COMWORK[i]+1]*group[i] +
alpha2_COMWORK[1,S0_COMWORK[i]+1]*(HRU_COMWORK0[i] - mean(HRU_COMWORK0[])) +
alpha3_COMWORK[1,S0_COMWORK[i]+1]*(EQ5D0[i] - mean(EQ5D0[]))
HRU_COMWORK1[i] ~dnorm(mu_COMWORK1[i], tau_COMWORK1[group[i]+1,S0_COMWORK[i]+1])
mu_COMWORK2[i] <- alpha0_COMWORK[2,S0_COMWORK[i]+1] + alpha1_COMWORK[2,S0_COMWORK[i]+1]*group[i] +
alpha2_COMWORK[2,S0_COMWORK[i]+1]*(HRU_COMWORK1[i] - mean(HRU_COMWORK1[])) +
alpha3_COMWORK[2,S0_COMWORK[i]+1]*(EQ5D1[i] - mean(EQ5D1[]))
HRU_COMWORK2[i] ~dnorm(mu_COMWORK2[i], tau_COMWORK2[group[i]+1,S0_COMWORK[i]+1])
mu_COMWORK0[i] <- alpha0_COMWORK[3,S0_COMWORK[i]+1]
HRU_COMWORK0[i] ~dnorm(mu_COMWORK0[S0_COMWORK[i]+1], tau_COMWORK0[S0_COMWORK[i]+1])

mu_GP1[i] <- alpha0_GP[1] + alpha1_GP[1]*group[i] + alpha2_GP[1]*(HRU_GP0[i] - mean(HRU_GP0[])) +
alpha3_GP[1]*(EQ5D0[i] - mean(EQ5D0[]))
HRU_GP1[i] ~dnorm(mu_GP1[i], tau_GP1[group[i]+1])
mu_GP2[i] <- alpha0_GP[2] + alpha1_GP[2]*group[i] + alpha2_GP[2]*(HRU_GP1[i] - mean(HRU_GP1[])) +
alpha3_GP[2]*(EQ5D1[i] - mean(EQ5D1[]))
HRU_GP2[i] ~dnorm(mu_GP2[i], tau_GP2[group[i]+1])
HRU_GP0[i] ~dnorm(mu_GP0, tau_GP0)

mu_NURSE1[i] <- alpha0_NURSE[1,S0_NURSE[i]+1] + alpha1_NURSE[1,S0_NURSE[i]+1]*group[i] +
alpha2_NURSE[1,S0_NURSE[i]+1]*(HRU_NURSE0[i] - mean(HRU_NURSE0[])) +
alpha3_NURSE[1,S0_NURSE[i]+1]*(EQ5D0[i] - mean(EQ5D0[]))
HRU_NURSE1[i] ~dnorm(mu_NURSE1[i], tau_NURSE1[group[i]+1,S0_NURSE[i]+1])
mu_NURSE2[i] <- alpha0_NURSE[2,S0_NURSE[i]+1] + alpha1_NURSE[2,S0_NURSE[i]+1]*group[i] +
alpha2_NURSE[2,S0_NURSE[i]+1]*(HRU_NURSE1[i] - mean(HRU_NURSE1[])) +
alpha3_NURSE[2,S0_NURSE[i]+1]*(EQ5D1[i] - mean(EQ5D1[]))
HRU_NURSE2[i] ~dnorm(mu_NURSE2[i], tau_NURSE2[group[i]+1,S0_NURSE[i]+1])
```

```
mu_NURSE0[i] <- alpha0_NURSE[3,S0_NURSE[i]+1]
HRU_NURSE0[i] ~dnorm(mu_NURSE0[S0_NURSE[i]+1], tau_NURSE0[S0_NURSE[i]+1])

mu_THERAP1[i] <- alpha0_THERAP[1,S0_THERAP[i]+1] + alpha1_THERAP[1,S0_THERAP[i]+1]*group[i] +
alpha2_THERAP[1,S0_THERAP[i]+1]*(HRU_THERAP0[i] - mean(HRU_THERAP0[])) +
alpha3_THERAP[1,S0_THERAP[i]+1]*(EQ5D0[i] - mean(EQ5D0[]))
HRU_THERAP1[i] ~dnorm(mu_THERAP1[i], tau_THERAP1[group[i]+1,S0_THERAP[i]+1])
mu_THERAP2[i] <- alpha0_THERAP[2,S0_THERAP[i]+1] + alpha1_THERAP[2,S0_THERAP[i]+1]*group[i] +
alpha2_THERAP[2,S0_THERAP[i]+1]*(HRU_THERAP1[i] - mean(HRU_THERAP1[])) +
alpha3_THERAP[1,S0_THERAP[i]+1]*(EQ5D1[i] - mean(EQ5D1[]))
HRU_THERAP2[i] ~dnorm(mu_THERAP2[i], tau_THERAP2[group[i]+1,S0_THERAP[i]+1])
mu_THERAP0[i] <- alpha0_THERAP[3,S0_THERAP[i]+1]
HRU_THERAP0[i] ~dnorm(mu_THERAP0[S0_THERAP[i]+1], tau_THERAP0[S0_THERAP[i]+1])

#models for S0s in the outcomes

S0_PSYDR[i] ~dbern(psi_S0PSYDR[i])
logit(psi_S0PSYDR[i]) <- gamma0_PSYDR + gamma1_PSYDR*group[i]

S0_PSYCH[i] ~dbern(psi_S0PSYCH[i])
logit(psi_S0PSYCH[i]) <- gamma0_PSYCH + gamma1_PSYCH*group[i]

S0_PHYSI[i] ~dbern(psi_S0PHYSI[i])
logit(psi_S0PHYSI[i]) <- gamma0_PHYSI + gamma1_PHYSI*group[i]

S0_DENT[i] ~dbern(psi_S0DENT[i])
logit(psi_S0DENT[i]) <- gamma0_DENT + gamma1_DENT*group[i]

S0_SOCWORK[i] ~dbern(psi_S0SOCWORK[i])
logit(psi_S0SOCWORK[i]) <- gamma0_SOCWORK + gamma1_SOCWORK*group[i]

S0_COMWORK[i] ~dbern(psi_S0COMWORK[i])
logit(psi_S0COMWORK[i]) <- gamma0_COMWORK + gamma1_COMWORK*group[i]

S0_NURSE[i] ~dbern(psi_S0NURSE[i])
logit(psi_S0NURSE[i]) <- gamma0_NURSE + gamma1_NURSE*group[i]

S0_THERAP[i] ~dbern(psi_S0THERAP[i])
logit(psi_S0THERAP[i]) <- gamma0_THERAP + gamma1_THERAP*group[i]

}

# parameter back-transformations

for(g in 1:2){
tau_PSYDR1[g,1] <- pow(sd_PSYDR1[g,1], -2)
tau_PSYDR2[g,1] <- pow(sd_PSYDR2[g,1], -2)
tau_PSYDR1[g,2] <- pow(sd_PSYDR1[g,2], -2)
tau_PSYDR2[g,2] <- pow(sd_PSYDR2[g,2], -2)
tau_PSYDR0[g] <- pow(sd_PSYDR0[g], -2)

tau_PSYCH1[g,1] <- pow(sd_PSYCH1[g,1], -2)
tau_PSYCH2[g,1] <- pow(sd_PSYCH2[g,1], -2)
tau_PSYCH1[g,2] <- pow(sd_PSYCH1[g,2], -2)
tau_PSYCH2[g,2] <- pow(sd_PSYCH2[g,2], -2)
tau_PSYCH0[g] <- pow(sd_PSYCH0[g], -2)

tau_PHYSI1[g,1] <- pow(sd_PHYSI1[g,1], -2)
tau_PHYSI2[g,1] <- pow(sd_PHYSI2[g,1], -2)
tau_PHYSI1[g,2] <- pow(sd_PHYSI1[g,2], -2)
tau_PHYSI2[g,2] <- pow(sd_PHYSI2[g,2], -2)
tau_PHYSI0[g] <- pow(sd_PHYSI0[g], -2)
```

```
tau_DENT1[g,1] <- pow(sd_DENT1[g,1], -2)
tau_DENT2[g,1] <- pow(sd_DENT2[g,1], -2)
tau_DENT1[g,2] <- pow(sd_DENT1[g,2], -2)
tau_DENT2[g,2] <- pow(sd_DENT2[g,2], -2)
tau_DENT0[g] <- pow(sd_DENT0[g], -2)

tau_SOCWORK1[g,1] <- pow(sd_SOCWORK1[g,1], -2)
tau_SOCWORK2[g,1] <- pow(sd_SOCWORK2[g,1], -2)
tau_SOCWORK1[g,2] <- pow(sd_SOCWORK1[g,2], -2)
tau_SOCWORK2[g,2] <- pow(sd_SOCWORK2[g,2], -2)
tau_SOCWORK0[g] <- pow(sd_SOCWORK0[g], -2)

tau_COMWORK1[g,1] <- pow(sd_COMWORK1[g,1], -2)
tau_COMWORK2[g,1] <- pow(sd_COMWORK2[g,1], -2)
tau_COMWORK1[g,2] <- pow(sd_COMWORK1[g,2], -2)
tau_COMWORK2[g,2] <- pow(sd_COMWORK2[g,2], -2)
tau_COMWORK0[g] <- pow(sd_COMWORK0[g], -2)

tau_NURSE1[g,1] <- pow(sd_NURSE1[g,1], -2)
tau_NURSE2[g,1] <- pow(sd_NURSE2[g,1], -2)
tau_NURSE1[g,2] <- pow(sd_NURSE1[g,2], -2)
tau_NURSE2[g,2] <- pow(sd_NURSE2[g,2], -2)
tau_NURSE0[g] <- pow(sd_NURSE0[g], -2)

tau_THERAP1[g,1] <- pow(sd_THERAP1[g,1], -2)
tau_THERAP2[g,1] <- pow(sd_THERAP2[g,1], -2)
tau_THERAP1[g,2] <- pow(sd_THERAP1[g,2], -2)
tau_THERAP2[g,2] <- pow(sd_THERAP2[g,2], -2)
tau_THERAP0[g] <- pow(sd_THERAP0[g], -2)

tau_GP1[g] <- pow(sd_GP1[g], -2)
tau_GP2[g] <- pow(sd_GP2[g], -2)

tau_EQ5D1[g] <- pow(sd_EQ5D1[g], -2)
tau_EQ5D2[g] <- pow(sd_EQ5D2[g], -2)
}
tau_GP0 <- pow(sd_GP0, -2)
tau_EQ5D0 <- pow(sd_EQ5D0, -2)

# priors on mean and std

mu_GP0 ~dnorm(0, 0.00001)
sd_GP0 ~dunif(0, 1000)

sd_EQ5D0 ~dunif(0, 1000)

for(g in 1:2){
sd_EQ5D1[g] ~dunif(0, 1000)
sd_EQ5D2[g] ~dunif(0, 1000)
}

# priors on level regression coefficients
for(l in 1:2){

beta0_EQ5D[l] ~dnorm (0, 0.0001)
beta1_EQ5D[l] ~dnorm (0, 0.0001)
beta2_EQ5D[l] ~dnorm (0, 0.0001)

alpha0_PSYDR[l,1] ~dnorm (0, 0.0001)
alpha1_PSYDR[l,1] ~dnorm (0, 0.0001)
alpha2_PSYDR[l,1] ~dnorm (0, 0.0001)
```

```
alpha3_PSYDR[l,1] ~dnorm (0, 0.0001)
alpha0_PSYDR[l,2] <- -1000000
alpha1_PSYDR[l,2] <- 0
alpha2_PSYDR[l,2] <- 0
alpha3_PSYDR[l,2] <- 0

alpha0_PSYCH[l,1] ~dnorm (0, 0.0001)
alpha1_PSYCH[l,1] ~dnorm (0, 0.0001)
alpha2_PSYCH[l,1] ~dnorm (0, 0.0001)
alpha3_PSYCH[l,1] ~dnorm (0, 0.0001)
alpha0_PSYCH[l,2] <- -1000000
alpha1_PSYCH[l,2] <- 0
alpha2_PSYCH[l,2] <- 0
alpha3_PSYCH[l,2] <- 0

alpha0_PHYSI[l,1] ~dnorm (0, 0.0001)
alpha1_PHYSI[l,1] ~dnorm (0, 0.0001)
alpha2_PHYSI[l,1] ~dnorm (0, 0.0001)
alpha3_PHYSI[l,1] ~dnorm (0, 0.0001)
alpha0_PHYSI[l,2] <- -1000000
alpha1_PHYSI[l,2] <- 0
alpha2_PHYSI[l,2] <- 0
alpha3_PHYSI[l,2] <- 0

alpha0_DENT[l,1] ~dnorm (0, 0.0001)
alpha1_DENT[l,1] ~dnorm (0, 0.0001)
alpha2_DENT[l,1] ~dnorm (0, 0.0001)
alpha3_DENT[l,1] ~dnorm (0, 0.0001)
alpha0_DENT[l,2] <- -1000000
alpha1_DENT[l,2] <- 0
alpha2_DENT[l,2] <- 0
alpha3_DENT[l,2] <- 0

alpha0_SOCWORK[l,1] ~dnorm (0, 0.0001)
alpha1_SOCWORK[l,1] ~dnorm (0, 0.0001)
alpha2_SOCWORK[l,1] ~dnorm (0, 0.0001)
alpha3_SOCWORK[l,1] ~dnorm (0, 0.0001)
alpha0_SOCWORK[l,2] <- -1000000
alpha1_SOCWORK[l,2] <- 0
alpha2_SOCWORK[l,2] <- 0
alpha3_SOCWORK[l,2] <- 0

alpha0_COMWORK[l,1] ~dnorm (0, 0.0001)
alpha1_COMWORK[l,1] ~dnorm (0, 0.0001)
alpha2_COMWORK[l,1] ~dnorm (0, 0.0001)
alpha3_COMWORK[l,1] ~dnorm (0, 0.0001)
alpha0_COMWORK[l,2] <- -1000000
alpha1_COMWORK[l,2] <- 0
alpha2_COMWORK[l,2] <- 0
alpha3_COMWORK[l,2] <- 0

alpha0_NURSE[l,1] ~dnorm (0, 0.0001)
alpha1_NURSE[l,1] ~dnorm (0, 0.0001)
alpha2_NURSE[l,1] ~dnorm (0, 0.0001)
alpha3_NURSE[l,1] ~dnorm (0, 0.0001)
alpha0_NURSE[l,2] <- -1000000
alpha1_NURSE[l,2] <- 0
alpha2_NURSE[l,2] <- 0
alpha3_NURSE[l,2] <- 0

alpha0_THERAP[l,1] ~dnorm (0, 0.0001)
alpha1_THERAP[l,1] ~dnorm (0, 0.0001)
```

```
alpha2_THERAP[l,1] ~dnorm (0, 0.0001)
alpha3_THERAP[l,1] ~dnorm (0, 0.0001)
alpha0_THERAP[l,2] <- -1000000
alpha1_THERAP[l,2] <- 0
alpha2_THERAP[l,2] <- 0
alpha3_THERAP[l,2] <- 0

alpha0_GP[l] ~dnorm (0, 0.0001)
alpha1_GP[l] ~dnorm (0, 0.0001)
alpha2_GP[l] ~dnorm (0, 0.0001)
alpha3_GP[l] ~dnorm (0, 0.0001)

}

beta0_EQ5D[3] ~dnorm (0, 0.0001)

for(l in 1:9){
beta_EQ5D0[l] ~dnorm(0, 0.0001)
beta_EQ5D1[l] ~dnorm(0, 0.0001)
beta_EQ5D2[l] ~dnorm(0, 0.0001)
}

alpha0_PSYDR[3,1] ~dnorm (0, 0.0001)
alpha0_PSYDR[3,2] <- -1000000
alpha0_PSYCH[3,1] ~dnorm (0, 0.0001)
alpha0_PSYCH[3,2] <- -1000000
alpha0_PHYSI[3,1] ~dnorm (0, 0.0001)
alpha0_PHYSI[3,2] <- -1000000
alpha0_DENT[3,1] ~dnorm (0, 0.0001)
alpha0_DENT[3,2] <- -1000000
alpha0_SOCWORK[3,1] ~dnorm (0, 0.0001)
alpha0_SOCWORK[3,2] <- -1000000
alpha0_COMWORK[3,1] ~dnorm (0, 0.0001)
alpha0_COMWORK[3,2] <- -1000000
alpha0_NURSE[3,1] ~dnorm (0, 0.0001)
alpha0_NURSE[3,2] <- -1000000
alpha0_THERAP[3,1] ~dnorm (0, 0.0001)
alpha0_THERAP[3,2] <- -1000000

gamma0_PSYDR ~dnorm(0, 0.0001)
gamma1_PSYDR ~dnorm(0, 0.0001)

gamma0_PSYCH ~dnorm(0, 0.0001)
gamma1_PSYCH ~dnorm(0, 0.0001)

gamma0_PHYSI ~dnorm(0, 0.0001)
gamma1_PHYSI ~dnorm(0, 0.0001)

gamma0_DENT ~dnorm(0, 0.0001)
gamma1_DENT ~dnorm(0, 0.0001)

gamma0_SOCWORK ~dnorm(0, 0.0001)
gamma1_SOCWORK ~dnorm(0, 0.0001)

gamma0_COMWORK ~dnorm(0, 0.0001)
gamma1_COMWORK ~dnorm(0, 0.0001)

gamma0_GP[1] ~dnorm(0, 0.0001)
gamma1_GP[1] ~dnorm(0, 0.0001)
gamma0_GP[2] ~dnorm(0, 0.0001)
gamma1_GP[2] ~dnorm(0, 0.0001)
gamma0_GP[3] ~dnorm(0, 0.0001)
```

```
gamma1_GP[3] ~dnorm(0, 0.0001)

gamma0_NURSE ~dnorm(0, 0.0001)
gamma1_NURSE ~dnorm(0, 0.0001)

gamma0_THERAP ~dnorm(0, 0.0001)
gamma1_THERAP ~dnorm(0, 0.0001)

} # end of the model block
```
